# Stability of synchronization in simplicial complexes

**DOI:** 10.1038/s41467-021-21486-9

**Published:** 2021-02-23

**Authors:** L. V. Gambuzza, F. Di Patti, L. Gallo, S. Lepri, M. Romance, R. Criado, M. Frasca, V. Latora, S. Boccaletti

**Affiliations:** 1 Department of Electrical, Electronics and Computer Science Engineering, University of Catania, Catania, Italy; 2 CNR-Institute of Complex Systems, Florence, Italy; 3 Department of Physics and Astronomy, University of Catania, Catania, Italy; 4 INFN Sezione di Catania, Catania, Italy; 5 Department of Applied Math. and Data, Complex Networks and Cybersecurity Research Institute, University Rey Juan Carlos, Madrid, Spain; 6 Istituto di Analisi dei Sistemi ed Informatica “A. Ruberti”, Consiglio Nazionale delle Ricerche (IASI-CNR), Roma, Italy; 7 School of Mathematical Sciences, Queen Mary University of London, London, UK; 8 The Alan Turing Institute, The British Library, London, UK; 9 Unmanned Systems Research Institute, Northwestern Polytechnical University, Xi’an, China; 10 Moscow Institute of Physics and Technology, Dolgoprudny, Moscow Region Russian Federation; 11 Universidad Rey Juan Carlos, Móstoles, Madrid Spain

**Keywords:** Complex networks, Nonlinear phenomena

## Abstract

Various systems in physics, biology, social sciences and engineering have been successfully modeled as networks of coupled dynamical systems, where the links describe pairwise interactions. This is, however, too strong a limitation, as recent studies have revealed that higher-order many-body interactions are present in social groups, ecosystems and in the human brain, and they actually affect the emergent dynamics of all these systems. Here, we introduce a general framework to study coupled dynamical systems accounting for the precise microscopic structure of their interactions at any possible order. We show that complete synchronization exists as an invariant solution, and give the necessary condition for it to be observed as a stable state. Moreover, in some relevant instances, such a necessary condition takes the form of a Master Stability Function. This generalizes the existing results valid for pairwise interactions to the case of complex systems with the most general possible architecture.

## Abstract (web summary)

Networks with higher order interactions, relevant to social groups, ecosystems and human brain, require new tools and instruments for their analysis. Gambuzza et al. propose an analytical approach which allows to find conditions for stable synchronization in many-body interaction networks.

## Introduction

Many systems in physics, biology, engineering and social sciences can be modeled as networks of interacting units^1^. Often, each of the elementary system constituents (the nodes of the network) is a dynamical system itself, whose evolution is influenced by the states of the other units to which is connected to through the links of the network. Unraveling how the interplay of network structure and the type of interactions shape the overall dynamics of the system and rule its collective behaviors is thus a problem of wide interest across disciplines.

There is an underlying strong assumption that is made when one adopts a network representation of a complex system: the overall interplay among the units of the system is assumed to be exhaustively described by combinations of pairwise interactions. Such an hypothesis may be justified when studying certain types of processes, but it is very short in representing faithfully other many circumstances. Indeed, from functional^2–4^ and structural^5^ brain networks to protein interaction networks^6^, to semantic networks^7^, random walks^8^ and co-authorship graphs in science^9^ there are a lot of practical situations which simply cannot be factorized in terms of pairwise interactions^10,11^.

Simplicial complexes are topological structures formed by simplices of different dimensions (such as nodes, links, triangles, tetrahedra, etc.) and map many-body interactions between the elements of a system. Differently from networks, simplicial complexes can therefore efficiently represent the interactions between any number of units. While simplicial complexes are not a new idea^12^, the availability of new data sets and the recent advances in topological data analysis techniques^13^ renewed the interest of the scientific community^14,15^. In particular, a lot of attention in the last years has been devoted to the modeling of simplicial complexes, and significant progresses were made in extending to simplicial complexes standard graph models, such as random graphs models^16^, the configuration model^17^, models of network growth^18^ and activity driven models^19^.

On the other hand, synchronization is a phenomenon appearing ubiquitously in natural and engineered systems^20–22^, and corresponds to the emergence of a collective behavior wherein the system units eventually adjust themselves into a common evolution in time. Various studies have shed light on the intimate relationships between the topology of a networked system, its synchronizability, and the properties of the synchronized states. In particular, synchronous behaviors have been observed and characterized in small-world ^23^, weighted^24^, multilayer^25^, and adaptive networks^26,27^. Outside complete synchronization, moreover, other types of synchronization have been revealed to emerge in networked systems, including remote synchronization^28,29^, cluster states^30^ and synchronization of group of nodes^31^, chimera^32,33^, Bellerophon states^34,35^, and Benjamin–Feir instabilities^36–38^. Finally, the transition to synchronization has been shown to be either smooth and reversible, or abrupt and irreversible (as in the case of explosive synchronization, resembling a first-order like phase transition^39^).

Extending the investigation of synchronization to structures including higher-order interactions is of great interest to many fields of study. An example is neuron dynamics where, on the one hand, synchronization plays a central role^40–43^ and, on the other hand, evidences of higher-order interactions between the neurons have been recently provided^44–46^. Ecological systems^47^ and nonlinear consensus^48^ constitute other examples where higher-order interactions may be fundamental in shaping the collective behavior of the system, thus further motivating such study.

While attempts of extending to *p*-uniform hypergraphs the analysis of complete synchronization of dynamical systems have been recently made^49^, the study of systems interplaying through higher order interactions in simplicial complexes has been so far limited to the case of the Kuramoto model^50,51^. This is, in fact, a specific model, wherein each unit of the ensemble *i* = 1, …, *N* is a phase oscillator and is characterized by the evolution of its real-valued phase *θ*_*i*_(*t*) ϵ [0, 2*π*]. The model has been studied in all different sorts of network topologies with possible applications to biological and social systems^21,50^, and recently extensions of it have been proposed that include higher-order interactions. Namely, it has been shown that the Kuramoto model may exhibit abrupt desynchronization when three-body interactions among all the oscillators are added to^52^, or completely replace^53^, the all-to-all pairwise interactions of the original model. Similar results have been obtained with a non-symmetric variation of the Kuramoto model in which the microscopic details of the interactions among the phase oscillators are described in the form of a simplicial complex^54^. A different approach has been proposed by Millán et al., who have formulated a higher-order Kuramoto model in which the oscillators are placed not on the nodes but on higher-order simplices, such as links, triangles, and so on, of a simplicial complex^55^. Finally, Lucas et al. have considered an extension of the Kuramoto model to high-order interactions of any order, which is still analytically tractable because all the oscillators have identical frequencies^56^.

We here abandon the limitation of sticking with a specific model system, and introduce instead the most general framework for the study of dynamical systems in simplicial complexes. Namely, we consider an ensemble of completely generic (yet identical) dynamical systems, organized on the nodes of a simplicial complex of generic order, and interacting via generic coupling functions. In other words, except for the fact that the systems have to be identical, we do not make any specific assumption that may limit in a way or another our approach. In such a wide context, we show that complete synchronization exists as an invariant solution as far as the coupling functions cancel out when nodes dynamics is identical. Furthermore, we give the necessary condition for it to be observed as a stable state, which in some instances takes the form of a Master Stability Function (MSF), a method initially developed in ref. ^57^ for pairwise coupled systems, and later extended in many ways to complex networks^58^ and to time-varying interactions^59–61^. Therefore, not only our framework includes and encompasses all studies made so far on the Kuramoto model, but it is valid for an enormously larger number of situations, and as so it is applicable to a very wide range of experimental and/or practical circumstances. We will show, indeed, that all the theoretical predictions that our method entitles us to make are fully verified in simulations of synthetic and real-word networked systems.

## Results

### Necessary condition for the synchronization of dynamical systems with higher-order interactions

The object of our study is the most general simplicial complex of *N* coupled dynamical units. This means that the different dynamical units are subject not only to pairwise interactions, but also to three-body interactions, four-body interactions and so on. The precise microscopic structure of the interactions is described by underlying simplicial complex, which can have any dimension *D* ≥ 1 (for all details and notations on simplicial complexes, see the “Methods”). In the particular case of *D* = 1, our system coincides with the standard case of a complex network of *N* coupled dynamical units. We assume that the equations of motion governing the dynamics of our *D*-dimensional simplicial complex can be written as:

1 $${\dot{{\bf{x}}}}_{i}=	 \, {\bf{f}}({{\bf{x}}}_{i})+{\sigma }_{1}\mathop{\sum }\limits_{{j}_{1}=1}^{N}{a}_{i{j}_{1}}^{(1)}{{\bf{g}}}^{(1)}({{\bf{x}}}_{i},{{\bf{x}}}_{{j}_{1}})\\ \, 	+{\sigma }_{2}\mathop{\sum }\limits_{{j}_{1}=1}^{N}\mathop{\sum }\limits_{{j}_{2}=1}^{N}{a}_{i{j}_{1}{j}_{2}}^{(2)}{{\bf{g}}}^{(2)}({{\bf{x}}}_{i},{{\bf{x}}}_{{j}_{1}},{{\bf{x}}}_{{j}_{2}})+\ldots \\ \, 	+{\sigma }_{D}\mathop{\sum }\limits_{{j}_{1}=1}^{N}...\mathop{\sum }\limits_{{j}_{D}=1}^{N}{a}_{i{j}_{1}....{j}_{D}}^{(D)}{{\bf{g}}}^{(D)}({{\bf{x}}}_{i},{{\bf{x}}}_{{j}_{1}},...,{{\bf{x}}}_{{j}_{D}}),$$

where **x**_*i*_(*t*) is the *m*-dimensional vector state describing the dynamics of unit *i*, *σ*_1_, …, *σ*_*D*_ are real-valued parameters describing coupling strengths, ${\bf{f}}:{{\mathbb{R}}}^{m}\longrightarrow {{\mathbb{R}}}^{m}$ describes the local dynamics (which is assumed identical for all units), while ${{\bf{g}}}^{(d)}:{{\mathbb{R}}}^{(d+1)\times m}\longrightarrow {{\mathbb{R}}}^{m}$ (*d* = 1, …, *D*) are synchronization noninvasive functions (i.e. **g**^(*d*)^(**x**, **x**, …, **x**) ≡ 0 ∀*d*) ruling the interaction forms at different orders. Furthermore, ${a}_{i{j}_{1}...{j}_{d}}^{(d)}$ are the entries of the adjacency tensors A^(*d*)^, with *d* = 1, …, *D*. These tensors, which generalize the notion of the adjacency matrix of a graph, describe the architecture of interactions of any order that can take place in the simplicial complex [see the “Methods” for a complete discussion on them and all quantities appearing in Eq. (1)]. This is the most general type of system we can consider, as there are no further specific restrictions on both the adjacency tensors of the simplicial complex and the functions **f** and **g**^(*d*)^.

As the notation may result somehow cumbersome, for the sake of clarity in what follows we illustrate the case of *D* = 2, so that a reader will be able to appreciate each and every conceptual action we are making. At the end, we will then summarize the steps one has to do in order to extrapolate the results to all values of *D*.

Let us then consider the following set of coupled differential equations

2 $${\dot{{\bf{x}}}}_{i}=	 \, {\bf{f}}({{\bf{x}}}_{i})+{\sigma }_{1}\mathop{\sum }\limits_{j=1}^{N}{a}_{ij}^{(1)}{{\bf{g}}}^{(1)}({{\bf{x}}}_{i},{{\bf{x}}}_{j})\\ \,	 +{\sigma }_{2}\mathop{\sum }\limits_{j=1}^{N}\mathop{\sum }\limits_{k=1}^{N}{a}_{ijk}^{(2)}{{\bf{g}}}^{(2)}({{\bf{x}}}_{i},{{\bf{x}}}_{j},{{\bf{x}}}_{k}),$$

where *σ*_1_ and *σ*_2_ are the coupling strengths associated to two- and three-body interactions.

Existence and invariance of the synchronized solution **x**^*s*^(*t*) = **x**_1_(*t*) = … = **x**_*N*_(*t*) are guaranteed by the noninvasiveness of the coupling functions. In order to study the stability of the synchronization solution, one considers small perturbations around the synchronous state, i.e., *δ***x**_*i*_ = **x**_*i*_ − **x**^*s*^, and perform a linear stability analysis of Eq. (2). To do this, one can perform the following transformation of the variables. Consider $\delta {\bf{x}}={[\delta {{\bf{x}}}_{1}^{T},\delta {{\bf{x}}}_{2}^{T},\ldots ,\delta {{\bf{x}}}_{N}^{T}]}^{T}$, and let us take, as a reference basis of ${{\mathbb{R}}}^{\,}$, the one made by the eigenvectors **v**_1_, **v**_2_, …, **v**_*N*_ of the classic Laplacian matrix ${{\mathcal{L}}}^{(1)}$. This allows to define new variables η = (V^−1^ ⊗ I_*m*_)δx, where *V* = [**v**_1_, **v**_2_, …, **v**_*N*_]. To express the dynamics of the system in terms of the new variables *η*, one needs to extend the notion of classical Laplacian matrix, which accounts for pairwise interactions, to a set of generalized Laplacian matrices, where the generic matrix of order *d*, indicated as ${{\mathcal{L}}}^{(d)}$, accounts for (*d* + 1)-body interactions (for a formal definition see “Methods”). In the specific case of *D* = 2, we will therefore describe the systems with two matrices, ${{\mathcal{L}}}^{(1)}$ and ${{\mathcal{L}}}^{(2)}$, respectively.

Through a series of three conceptual steps detailed in the “Methods”, the following equations can be derived

3 $$\left\{\begin{array}{lll}{\dot{{\eta }}}_{1}={\rm{JF}}{{{\eta }}}_{1}\hfill\\ \dot{{{\eta }}_{i}}=({\rm{JF}}-{\sigma }_{1}{\lambda }_{i}{{\rm{JG}}}^{(1)}){{\eta }}_{i}-{\sigma }_{2}\mathop{\sum }\limits_{j=2}^{N}{\tilde{{\mathcal{L}}}}_{ij}^{(2)}{{\rm{JG}}}^{(2)}{{\eta }}_{j},\end{array}\right.$$

where JF = *J***f**(**x**^*s*^), JG^(1)^ = *J***g**^(1)^(**x**^*s*^, **x**^*s*^) and JG^(2)^ = *J*_1_**g**^(2)^(**x**^*s*^, **x**^*s*^, **x**^*s*^) + *J*_2_**g**^(2)^(**x**^*s*^, **x**^*s*^, **x**^*s*^) represent the Jacobian matrices for the functions **f**, **g**^(1)^ and **g**^(2)^ respectively, 0 = *λ*_1_ < *λ*_2_ ≤…*λ*_*N*_ are the eigenvalues of ${{\mathcal{L}}}^{(1)}$, and ${\tilde{{\mathcal{L}}}}_{ij}^{(2)}$ are suitable, known, coefficients given by transforming ${{\mathcal{L}}}^{(2)}$ with the matrix *V* that diagonalizes the classic Laplacian ${{\mathcal{L}}}^{(1)}$ (see the “Methods” for all details). The dynamics of the linearized system is then decoupled into two parts: the dynamics of *η*_1_, accounting for the motion along the synchronous manifold, and that of all other variables η_*i*_ (with *i* = 2, …, *N*), representing the different modes transverse to the synchronization manifold, and coupled each other by means of the coefficients ${\tilde{{\mathcal{L}}}}_{ij}^{(2)}$ (all of them being known quantities).

The problem of stability is then reduced to: (i) simulating a single, uncoupled, nonlinear system; (ii) using the obtained trajectory to feed up the elements of the Jacobians JG^(1)^ and JG^(2)^; (iii) simulating the dynamics of a system of *N* − 1 coupled linear equations, and tracking the behavior of the norm $\sqrt{\mathop{\sum }\nolimits_{i = 2}^{N}\mathop{\sum }\nolimits_{j = 1}^{m}{({\eta }_{i}^{(j)})}^{2}}$ for the calculation of the maximum Lyapunov exponent (being ${{\eta }}_{i}\equiv ({\eta }_{i}^{(1)},{\eta }_{i}^{(2)},...,{\eta }_{i}^{(m)})$).

Stability of the synchronous solution requires as a necessary condition that Λ_max_, the maximum among the (conditional) Lyapunov exponents associated to all transverse modes, be negative. Given the node dynamics and the coupling functions, Λ_max_ is in general function of the topology of the two-body interactions, the topology of the three body interactions, and the two coupling strengths *σ*_1_ and *σ*_2_, i.e., Λ_max_ = ${{{\Lambda }}}_{\rm{max}}({\sigma }_{1},{\sigma }_{2},{{\mathcal{L}}}^{(1)},{{\mathcal{L}}}^{(2)})$.

It is important to notice that, in analogy with the classical MSF approach, also in the case of simplicial complexes one is, therefore, able to separate the motion along the synchronization manifold and that transverse to it, and such a crucial separation ultimately enables the study of stability of the synchronous manifold. For simplicial complexes, however, the higher complexity in the structure of the interactions yields a formalism requiring the analysis of a set of coupled differential equations, rather than of a single parametric variational equation (as in the case of the MSF). In other words, in the fully general case the set of equations describing the motion transverse to the synchronous manifold cannot be further decomposed into independent, decoupled modes, as it happens in the network case; however, the analysis of stability still requires a straightforward computation of a single quantity, i.e., the maximum Lyapunov exponent, which has to be performed on such a set of coupled, linear equations. In the more general case, the transverse modes are intertwined, such that stability has to be analyzed without reduction in dimensionality. However, we will momentarily show that there are relevant instances where such an expression can be simplified, up to recover a formalism that is identical to the classical MSF, allowing separation of the modes and reduction of the dimensionality of the problem to a single parameteric variational equation.

**Fig. 1:**
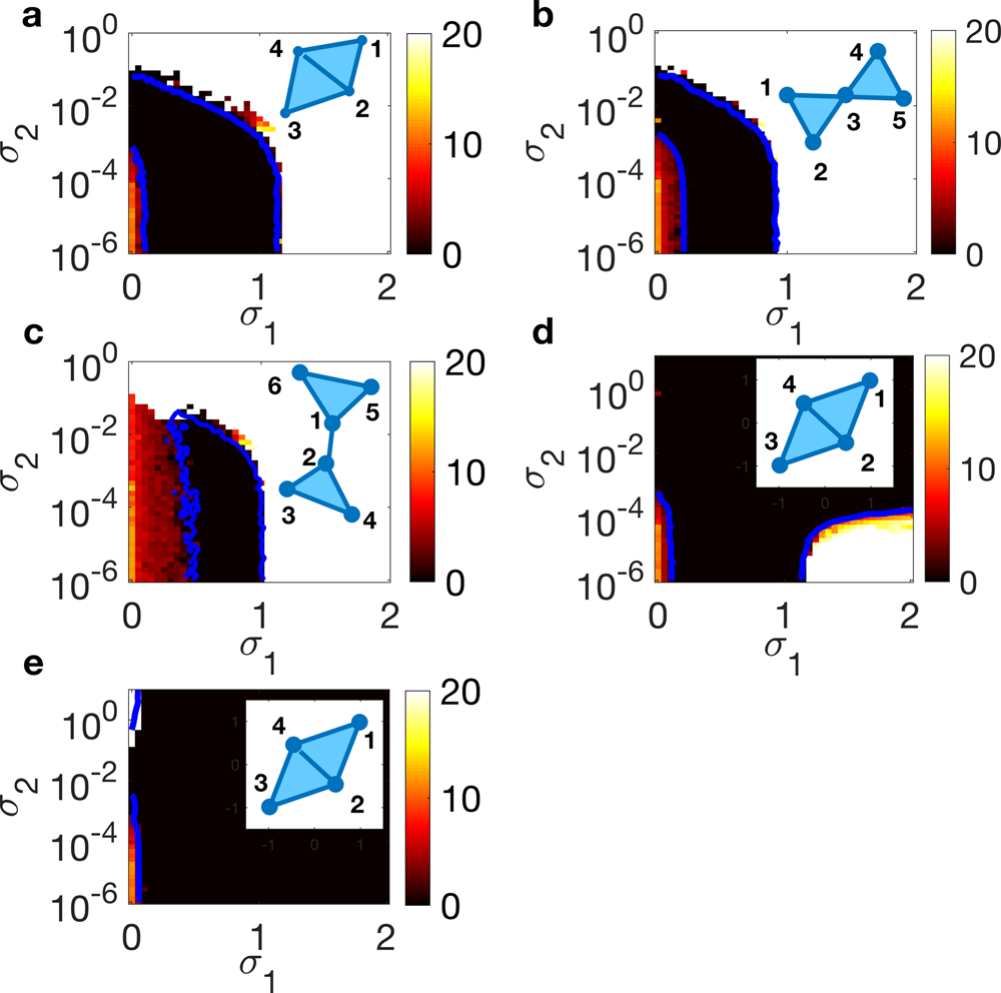
Synchronization in simplicial complexes of Rössler oscillators. Contour plots of the time averaged (over an observation time *T* = 500) synchronization error *E* (see “Methods” for definition and the vertical bars of each panel for the color code) in the plane (*σ*_1_, *σ*_2_) for some examples of simplicial complexes (whose sketches are reported in the top left of each panel). Simulations refer to coupled Rössler oscillators (**x** = (*x*, *y*, *z*)^*T*^ and **f** = (−*y*−*z*, *x*+*ay*, *b*+*z*(*x*−*c*))^*T*^) with parameters fixed in the chaotic regime (*a* = *b* = 0.2, *c* = 9). In **a**–**d**, ${{\bf{g}}}^{(1)}({{\bf{x}}}_{i},{{\bf{x}}}_{j})={[{x}_{j}-{x}_{i},0,0]}^{T}$, while in (**e**) ${{\bf{g}}}^{(1)}({{\bf{x}}}_{i},{{\bf{x}}}_{j})={[0,{y}_{j}-{y}_{i},0]}^{T}$. As for the other coupling function, one has ${{\bf{g}}}^{(2)}({{\bf{x}}}_{i},{{\bf{x}}}_{j},{{\bf{x}}}_{k})={[0,{y}_{j}^{2}{y}_{k}-{y}_{i}^{3},0]}^{T}$ in (**d**) and ${{\bf{g}}}^{(2)}({{\bf{x}}}_{i},{{\bf{x}}}_{j},{{\bf{x}}}_{k})={[{x}_{j}^{2}{x}_{k}-{x}_{i}^{3},0,0]}^{T}$ in all other panels. The blue continuous lines are the theoretical predictions of the synchronization thresholds obtained from Eq. (3). **a**, **b**, and **c** are examples of class III problems, whereas panels **d** and **e** are examples of class II problems.

In analogy with the classification of systems made for synchronization of complex networks (Chapter 5 in ref. ^1^), one immediately realizes that, once specified the dynamical system taking place in each node (i.e., the function **f**), the various coupling functions **g**^(1)^ and **g**^(2)^, and the structure of the simplicial complex (i.e., ${{\mathcal{L}}}^{(1)}$ and ${{\mathcal{L}}}^{(2)}$), all possible cases can be divided in three classes: (i) class I problems, where Λ_max_ is positive in all the half plane (*σ*_1_ ≥ 0, *σ*_2_ ≥ 0), and therefore synchronization is never stable; (ii) class II problems, for which Λ_max_ is negative within a unbounded area of the half plane (*σ*_1_ ≥ 0, *σ*_2_ ≥ 0); and (iii) class III problems, for which the area of the half plane (*σ*_1_ ≥ 0, *σ*_2_ ≥ 0) in which Λ_max_ is negative is instead bounded, and therefore additional instabilities of the synchronous motion may occur at larger values of the coupling strengths. While class I problems are trivial (in that synchronization is never observed), examples of class II and class III problems are shown in Fig. 1 for simplicial complexes of Rössler oscillators^62^, and one easily sees that the predictions made by solving Eq. (3) are indeed fully confirmed by the simulations of the original system in Eq. (2).

Far from being limited to the case of *D* = 2, our approach can be extended straightforwardly to simplicial complexes of any order *D*. Each term on the right hand side of Eq. (1) can, indeed, be manipulated following exactly the same three conceptual steps described in the “Methods”. Once again, one is entitled to select the eigenvector set which diagonalizes ${{\mathcal{L}}}^{(1)}$, to introduce the new variables *η* = (*V*^−1^ ⊗ *I*_*m*_)*δ***x**. Following the very same steps which led us to write Eq. (3), one then obtains

4 $${\dot{{\eta }}}_{1} 	= \, {\rm{JF}}{{\eta }}_{1},\\ \dot{{{\eta }}_{i}}	= \, ({\rm{JF}}-{\sigma }_{1}{\lambda }_{i}{{\rm{JG}}}^{(1)}){{\eta }}_{i}-{\sigma }_{2}\mathop{\sum }\limits_{j=2}^{N}{\tilde{{\mathcal{L}}}}_{ij}^{(2)}{{\rm{JG}}}^{(2)}{{\eta }}_{j}-\ldots \\ \,	\quad-{\sigma }_{D}\mathop{\sum }\limits_{j=2}^{N}{\tilde{{\mathcal{L}}}}_{ij}^{(D)}{{\rm{JG}}}^{(D)}{{\eta }}_{j},$$

where JG^(*d*)^  =  *J*_1_**g**^(*d*)^(**x**^*s*^, …, **x**^*s*^)  +  *J*_2_**g**^(*d*)^(**x**^*s*^, …, **x**^*s*^) +…+ *J*_*d*_**g**^(*d*)^(**x**^*s*^, …, **x**^*s*^) and the coefficients ${\tilde{{\mathcal{L}}}}_{ij}^{(d)}$ result from transforming ${{\mathcal{L}}}^{(d)}$ with the matrix that diagonalizes ${{\mathcal{L}}}^{(1)}$. As a result, one has conceptually the same reduction of the problem to a single, uncoupled, nonlinear system, plus a system of *N* − 1 coupled linear equations, from which the maximum Lyapunov exponent Λ_max_ = ${{{\Lambda }}}_{\rm{max}}({\sigma }_{1},{\sigma }_{2},...,{\sigma }_{D},{{\mathcal{L}}}^{(1)},{{\mathcal{L}}}^{(2)},...,{{\mathcal{L}}}^{(D)})$ can be extracted and monitored (for each simplicial complex) in the *D*-dimensional hyper-space of the coupling strength parameters.

### The MSF for synchronization in simplicial complexes

Our results can be greatly simplified in a series of relevant cases in which either the topology of the connectivity structure, or the coupling functions, allow to formulate our approach in terms of MSF. Once again, for the sake of illustration, we will start considering first the case of *D* = 2, and then the extension to any order *D*.

The first case is an all-to-all coupling, for which every two and three-body interaction is active. In this case, the classical Laplacian matrix is.

5 $${{\mathcal{L}}}_{ij}^{(1)}=\left\{\begin{array}{lll} -1 \hfill&\,{\text{for}}\,& i\,\ne\, j\hfill\\ N-1 &\,{\text{for}}\,& i=j.\end{array}\right.$$

Then, it is easy to rewrite ${{\mathcal{L}}}^{(2)}$, because the off diagonal terms ${{\mathcal{L}}}_{ij}^{(2)}$ (*i* ≠ *j*) represent the number of triangles formed by the link (*i*, *j*) which, in the present case, is simply equal to *N* − 2. Second, we consider the terms of the main diagonal ${{\mathcal{L}}}_{ii}^{(2)}$, the number of triangles having the node *i* as a vertex, which is

6 $${k}_{i}^{(2)}=\left(\begin{array}{c}N-1\\ 2\end{array}\right)=\frac{(N-1)(N-2)}{2}.$$

Consequently, one has that

7 $${{\mathcal{L}}}^{(2)}=(N-2)\ {{\mathcal{L}}}^{(1)}.$$

Starting from Eq. (2), applying the steps detailed in the Methods and noticing that in the all-to-all configuration *λ*_2_ = …*λ*_*N*_ = *N*, for each **η**_*i*_ (with $i\in \left\{2,\ldots ,N\right\}$), one obtains

8 $${\dot{{\eta }}}_{i}=[{\rm{JF}}-{\sigma }_{1}N{{\rm{JG}}}^{(1)}-{\sigma }_{2}N(N-2){{\rm{JG}}}^{(2)}]{{\eta }}_{i}.$$

In other words, in the all-to-all case, the variables *η*_*i*_ come out to be all uncoupled to each other, so that Λ_max_ uniquely depends on *σ*_1_, *σ*_2_ and *N*, i.e., Λ_max_ = Λ_max_(*σ*_1_, *σ*_2_, *N*).

In the more general case of a *D*-dimensional simplicial complex, it is easy to write the generalized Laplacian of order *d* as a function of the classical Laplacian matrix. In fact, the number of *d*-simplices having node *i* as a vertex and the number of *d*-simplices formed by the link (*i*, *j*) are respectively

9 $${k}_{i}^{(d)}=\left(\begin{array}{c}N-1\\ d\end{array}\right)=\frac{(N-1)(N-2)\ldots (N-d)}{d!}$$

and

10 $${k}_{ij}^{(d)}=\left(\begin{array}{c}N-2\\ d-1\end{array}\right)=\frac{(N-2)\ldots (N-d)}{(d-1)!}.$$

Given the definition of the generalized Laplacian, we find that

11 $${{\mathcal{L}}}^{(d)}	= \, (N-d)\ {{\mathcal{L}}}^{(d-1)}\\ 	= \, (N-2)(N-3)\ldots (N-d){{\mathcal{L}}}^{(1)}.$$

Once again, one can derive a parametric equation analogous to Eq. (8), with a MSF (once fixed both the node dynamics and the coupling functions) which solely depends on the coupling coefficients and the number of nodes, i.e. Λ_max_ = Λ_max_(*σ*_1_, *σ*_2_, …, *σ*_*D*_, *N*)

12 $${\dot{{\eta }}}_{i}=	 \, \left[{\rm{JF}}-{\sigma }_{1}N{{\rm{JG}}}^{(1)}-{\sigma }_{2}N(N-2){{\rm{JG}}}^{(2)}-\ldots \ \right.\\ \, 	\left.-{\sigma }_{D}N(N-2)\ldots (N-D){{\rm{JG}}}^{(D)}\right]{{\eta }}_{i}.$$

Another interesting case is that of generalized diffusion interactions with natural coupling functions. This amounts to consider diffusive coupling functions, given by

13 $${{\bf{g}}}^{(1)}({{\bf{x}}}_{i},{{\bf{x}}}_{j})	= \, {{\bf{h}}}^{(1)}({{\bf{x}}}_{j})-{{\bf{h}}}^{(1)}({{\bf{x}}}_{i}),\\ {{\bf{g}}}^{(2)}({{\bf{x}}}_{i},{{\bf{x}}}_{j},{{\bf{x}}}_{k})	= \, {{\bf{h}}}^{(2)}({{\bf{x}}}_{j},{{\bf{x}}}_{k})-{{\bf{h}}}^{(2)}({{\bf{x}}}_{i},{{\bf{x}}}_{i}),$$

where ${{\bf{h}}}^{(1)}:{{\mathbb{R}}}^{m}\longrightarrow {{\mathbb{R}}}^{m}$ and ${{\bf{h}}}^{(2)}:{{\mathbb{R}}}^{2m}\longrightarrow {{\mathbb{R}}}^{m}$. In addition, a condition of natural coupling is considered:

14 $${{\bf{h}}}^{(2)}({\bf{x}},{\bf{x}})={{\bf{h}}}^{(1)}({\bf{x}}).$$

Equation (14) expresses, indeed, the fact that the coupling to node *i* from two-body and three-body interactions is essentially similar, in that a three-body interaction where two nodes are on the same state is equivalent to a two-body interaction. Here, our approach takes the form of a MSF with a particularly convenient expression, as it can be written as a function of a single parameter. In fact, in this case, the transverse modes can be fully decoupled (see the “Methods” for the full derivation) and a single parameter MSF can be defined, starting from the following *m*-dimensional linear parametric variational equation

15 $$\dot{{\eta }}=\left[J{\bf{f}}({{\bf{x}}}^{s})-\alpha J{{\bf{h}}}^{(1)}({{\bf{x}}}^{s})\right]{\eta }$$

from which the maximum Lyapunov exponent is calculated: Λ_max_ = Λ_max_(*α*) with $\alpha =\lambda ({\sigma }_{1}{{\mathcal{L}}}^{(1)}+{\sigma }_{2}{{\mathcal{L}}}^{(2)})$ or $\alpha ={\sigma }_{1}\lambda ({{\mathcal{L}}}^{(1)}+r{{\mathcal{L}}}^{(2)})={\sigma }_{1}\lambda ({\mathcal{M}})$, where ${\mathcal{M}}$ is given by ${{\mathcal{L}}}^{(1)}+r{{\mathcal{L}}}^{(2)}$ with $r=\frac{{\sigma }_{2}}{{\sigma }_{1}}$. The situation, is therefore, conceptually equivalent to that of synchronization in complex networks, with the effective matrix ${\mathcal{M}}$ playing the same role of the classical Laplacian: given the dynamical system **f**, the coupling functions **h**^(1)^ and **h**^(2)^, and the structure of connection of the simplicial complex (i.e., ${{\mathcal{L}}}^{(1)}$ and ${{\mathcal{L}}}^{(2)}$) one can define three possible classes of problems:

(i) class I problems, for which the curve Λ_max_ = Λ_max_(*α*) does not intercept the abscissa and it is always positive. In this case synchronization is always forbidden, no matter which simplicial complex is used for connecting the dynamical systems;
(ii) class II problems, for which the curve Λ_max_ = Λ_max_(*α*) intercepts the abscissa only once at *α*_c_, and for which, therefore, the synchronization threshold is given by the self consistent equation ${\sigma }_{1}^{\rm{critical}}={\alpha }_{c}/{\lambda }_{2}[{\mathcal{M}}({\sigma }_{1}^{\rm{critical}},{\sigma }_{2}^{\rm{critical}})]$, i.e. it scales with the inverse of the second smallest eigenvalue of the effective matrix;
(iii) class III problems, for which the curve Λ_max_ = Λ_max_(*α*) intercepts the abscissa twice at *α*_1_ and *α*_2_ > *α*_1_. In this case, synchronization can be observed only if the entire eigenvalue spectrum of the effective matrix is such that ${\sigma }_{1}{\lambda }_{2}({\mathcal{M}})> {\alpha }_{1}$ and, at the same time, ${\sigma }_{1}{\lambda }_{N}({\mathcal{M}})<{\alpha }_{2}$. In this case, the parameter $\frac{{\lambda }_{2}({\mathcal{M}})}{{\lambda }_{N}\left({\mathcal{M}}\right)}$ can be considered as a proxy measure of synchronizability of the simplicial complex, in that the closer is such a parameter to unity (the more compact is the spectrum of eigenvalue of ${\mathcal{M}}$) the larger can be the range of coupling strengths for which the two above synchronization conditions can be satisfied.

We have so far considered the case of *D* = 2. In the fully general scenario, the condition for natural coupling is given by

16 $${{\bf{h}}}^{(D)}({\bf{x}},\ldots ,{\bf{x}})=\ldots ={{\bf{h}}}^{(2)}({\bf{x}},{\bf{x}})={{\bf{h}}}^{(1)}({\bf{x}}).$$

The equation for the MSF is formally analogous to Eq. (15), where now $\alpha ={\sigma }_{1}{\lambda }({{\mathcal{M}}}^{(D)})$ parameterizes the eigenvalues of the effective matrix of order *D*

17 $${{\mathcal{M}}}^{(D)}={{\mathcal{L}}}^{(1)}+\frac{{\sigma }_{2}}{{\sigma }_{1}}{{\mathcal{L}}}^{(2)}+\ldots +\frac{{\sigma }_{D}}{{\sigma }_{1}}{{\mathcal{L}}}^{(D)}.$$

In summary, we have shown that, while in the general case the transverse modes are intertwined, in the case of all-to-all coupling or of natural coupling functions a significant dimensionality reduction of the stability analysis problem is obtained, through the formulation of a MSF (Eq. (12) for all-to-all coupling and Eq. (15) for natural coupling).

### Synchronization in simplicial complexes of chaotic systems

Following is a series of results confirming the validity and wide applicability of our approach. We focus on two paradigmatic three-dimensional (${\bf{x}}={(x,y,z)}^{T}\in {{\mathbb{R}}}^{3}$) chaotic systems, namely the Rössler oscillator^62^ and the Lorenz system^63^, and, as a real-world example of neuron dynamics, on the Hindmarsh-Rose (HR) model^64^ (see the “Methods” for the equations describing the three systems, as well as for the setting of parameters and of stipulations for the numerical simulations). In particular, we start with considering the more general case with diffusive coupling, then we discuss our results on neuron dynamics and on the MSF cases of all-to-all and natural coupling, where we also show an analysis carried out on a real-world structure. Finally, we move away from the study of complete synchronization and illustrate an example of cluster synchronization in simplicial complexes.

### The general case

Our discussion begins with going back to Fig. 1, where we have considered a few elementary configurations of simplicial complexes, chosen in order to illustrate the classes of problems that one can deal with even when the structures involve only a small number of nodes. In particular, Fig. 1 reveals that synchronization in the general case crucially depends on the topology and the coupling functions: the same configuration can in fact feature different dynamics when diverse mechanisms regulate the coupling and, conversely, the same coupling functions may lead to different behaviors when the topology of interactions changes.

As an example, let us consider the full dynamical equations of coupled Rössler oscillators, when the coupling functions are chosen as ${{\bf{g}}}^{(1)}({{\bf{x}}}_{i},{{\bf{x}}}_{j})={[{x}_{j}-{x}_{i},0,0]}^{T}$ and ${{\bf{g}}}^{(2)}({{\bf{x}}}_{i},{{\bf{x}}}_{j},{{\bf{x}}}_{k})={[{x}_{j}^{2}{x}_{k}-{x}_{i}^{3},0,0]}^{T}$. They read

18 $${\dot{x}}_{i}=	 \, -{y}_{i}-{z}_{i}+{\sigma }_{1}\mathop{\sum }\limits_{j=1}^{N}{a}_{ij}^{(1)}({x}_{j}-{x}_{i})\\ \, 	+{\sigma }_{2}\mathop{\sum }\limits_{j=1}^{N}\mathop{\sum }\limits_{k=1}^{N}{a}_{ijk}^{(2)}({x}_{j}^{2}{x}_{k}-{x}_{i}^{3}),\\ {\dot{y}}_{i}	= \, {x}_{i}+a{y}_{i},\\ {\dot{z}}_{i}	= \, b+{z}_{i}({x}_{i}-c),$$

In each of the configurations considered, the state of the system is monitored by the average synchronization error *E* defined in the “Methods”. Figure 1 reports *E*(*σ*_1_, *σ*_2_) for different simplicial complexes (shown as insets in the panels) and coupling functions, along with the theoretical predictions provided by Eq. (3) (the blue, continuous, lines superimposed to the diagrams of the synchronization error). In all the cases, the numerical simulations are in very good agreement with the theoretical predictions for the synchronization thresholds.

The results of Fig. 1 suggest several interesting considerations. Indeed, in the cases reported in panels a and b of Fig. 1 synchronization may be achieved using either two-body or three-body interactions only (for very small *σ*_1_ indeed there is a range of values of *σ*_2_ leading to synchronization, and viceversa), while in the case of panel c synchronization is forbidden for very small values of *σ*_1_. In the last case, in fact, the two triangles do not have a common edge as in Fig. 1a, nor a common node as in Fig. 1b, and therefore interactions through links becomes essential for synchronization. Finally, one notice that there are scenarios, as in panels d and e, where the synchronization region is unbounded. As already mentioned, Fig. 1 provides examples of two of the three possible classes of behavior, with class III behavior in Fig. 1a–c, and class II in Fig. 1d (where synchronization exists in an unbounded region of the coupling coefficient regulating pairwise interactions, i.e., *σ*_1_) and in Fig. 1e (where synchronization exists in an unbounded region of the coupling coefficient regulating three-body interactions, i.e., *σ*_2_).

### Applications to neuron dynamics

We now discuss the applicability of our framework to the study of neuron dynamics. Synchronization in neural activity is of utmost importance. On the one hand, synchronized network oscillations are known to play a role in establishing ensembles of neurons in a task-dependent, flexible manner^40^. On the other hand, synchronization of neural activity is associated to epileptic seizures^41–43^. Recent evidences in neuroscience have pointed out the existence of higher-order interactions between neurons^44^. In particular, astrocytes and other glial cells are considered a plausible biological source of high-order interactions^45,46^, as they make contact with thousands of synapses and actively modulate their function^65^. Although how to account for these interactions in nonlinear models of neuron dynamics is still an open problem, here we discuss an example showing the suitability of our framework to study neuronal synchronization in the presence of high-order coupling. As a pratical case study, we here consider an ensemble of HR neurons, subject not only to pairwise coupling but also to three-body interactions. The system is described by

19 $${\dot{x}}_{i}	= \, {y}_{i}+3{x}_{i}^{2}-{x}_{i}^{3}-{z}_{i}+I+{\sigma }_{1}\mathop{\sum }\limits_{j=1}^{N}{a}_{ij}^{(1)}\tanh \left(\frac{{x}_{j}-{x}_{i}}{0.5}\right)\\ \, 	\quad+{\sigma }_{2}\mathop{\sum }\limits_{j=1}^{N}\mathop{\sum }\limits_{k=1}^{N}{a}_{ijk}^{(2)}\tanh \left(\frac{{x}_{j}+{x}_{k}-2{x}_{i}}{0.5}\right),\\ {\dot{y}}_{i}	= \, 1-5{x}_{i}^{2}-{y}_{i},\\ {\dot{z}}_{i}	= \, -r{z}_{i}+rs({x}_{i}+1.6),$$

where the non-diffusive coupling functions on the membrane potential account for possible saturation phenomena. Figure 2 shows the results, representing further examples of class II problems, where synchronization in neuronal activity is achieved in an unbounded region of the coupling coefficients *σ*_1_ and *σ*_2_. We notice that, in this case, three-body interactions are beneficial for synchronization as they lower the value of the pairwise coupling strength needed to achieve it.

**Fig. 2:**
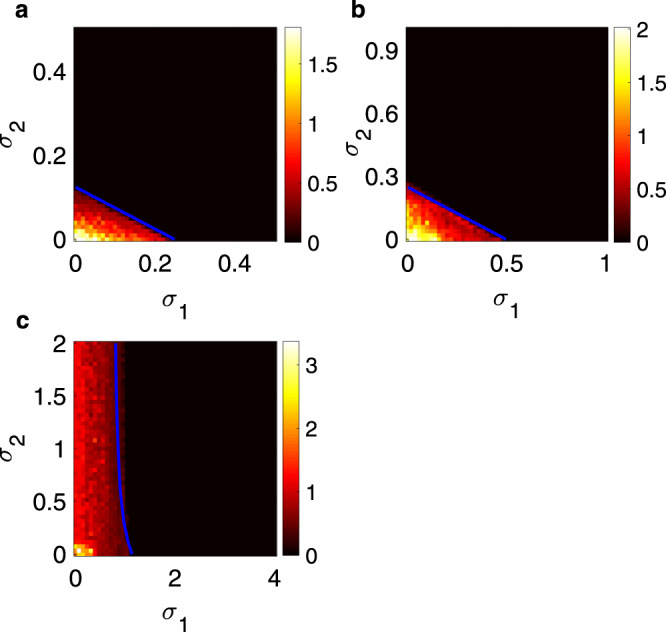
Synchronization in simplicial complexeses of Hindmarsh–Rose neurons. Contour plots of the time averaged (over an observation time *T* = 500) synchronization error *E* (see “Methods” for definition and the vertical bars of each panel for the color code) in the plane (*σ*_1_, *σ*_2_) for simplicial complexes of HR neurons coupled as in Eq. (19). Parameters are fixed in the chaotic regime (*r* = 0.006, *s* = 4, *I* = 3.2). **a**–**c** refer to three different simplicial complexes corresponding to the structures considered in Fig. 1a–c. The blue continuous lines are the theoretical predictions of the synchronization thresholds obtained from Eq. (3).

### MSF cases

Let us now move to discuss other results, which refer to the cases where our approach yields a MSF. We start with the all-to-all coupling case where, according to Eq. (8), one obtains a MSF that is function of *N*, *σ*_1_ and *σ*_2_. We then consider a simplicial complex of Rössler oscillators with all-to-all coupling, described by

20 $${\dot{x}}_{i}=	 \, -{y}_{i}-{z}_{i}+{\sigma }_{1}\mathop{\sum }\limits_{j=1}^{N}({x}_{j}-{x}_{i})+{\sigma }_{2}\mathop{\sum }\limits_{j=1, j\neq i}^{N}\mathop{\sum }\limits_{k=1, k\neq i}^{N}({x}_{j}^{2}{x}_{k}-{x}_{i}^{3}),\\ {\dot{y}}_{i}=	 \, {x}_{i}+a{y}_{i},\\ {\dot{z}}_{i}=	 \, b+{z}_{i}({x}_{i}-c).$$

**Fig. 3:**
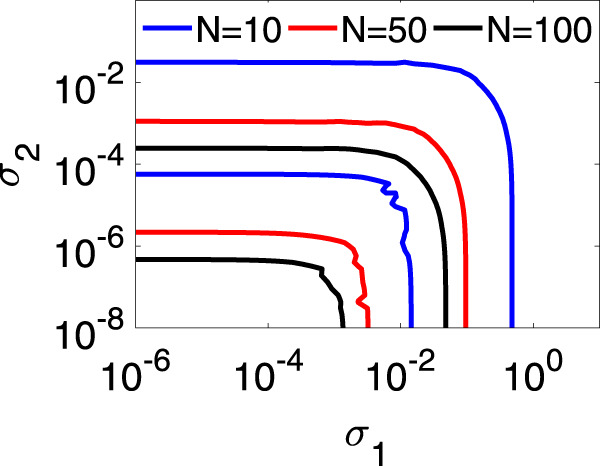
Synchronization in a simplicial complex of Rössler oscillators with all-to-all coupling. Lower and upper boundary curves for the region where synchronization is stable, at different values of *N*. The color codes for the different curves is reported at the top of the panel.

The results are shown in Fig. 3 for three values of *N* (*N* = 10, *N* = 50, and *N* = 100): the synchronous manifold is stable in a bounded region of the semiplane (*σ*_1_ > 0, *σ*_2_ > 0) delimited by blue (*N* = 10), red (*N* = 50) and black (*N* = 100) lines. One immediately sees that such a stability region moves toward the origin when *N* is increased. Hence, increasing *N* reduces the lower and upper thresholds for achieving synchronization.

Finally, we consider the case of natural coupling. Here, in full analogy with what occurs for networks, the MSF is a function of a single parameter, i.e., Λ_max_ = Λ_ma*x*_(*α*) with $\alpha =\lambda ({\sigma }_{1}{{\mathcal{L}}}^{(1)}+{\sigma }_{2}{{\mathcal{L}}}^{(2)})$ or $\alpha ={\sigma }_{1}\lambda ({{\mathcal{L}}}^{(1)}+r{{\mathcal{L}}}^{(2)})={\sigma }_{1}\lambda ({\mathcal{M}})$. This enables the study of synchronization stability into two steps, one pertaining only to the node dynamics and coupling functions, providing Λ_max_ = Λ_max_(*α*), and a second step, where the condition Λ_max_(*α*) < 0 is checked at the points $\alpha =\{{\sigma }_{1}{\lambda }_{2}({\mathcal{M}}),\ldots ,{\sigma }_{1}{\lambda }_{N}({\mathcal{M}})\}$.

We calculated the MSF for the Rössler oscillator and the Lorenz system with several choices of the coupling functions: ${{\bf{h}}}^{(1)}({{\bf{x}}}_{j})={[{x}_{j}^{3},0,0]}^{T}$ and ${{\bf{h}}}^{(2)}({{\bf{x}}}_{j},{{\bf{x}}}_{k})={[{x}_{j}^{2}{x}_{k},0,0]}^{T}$; ${{\bf{h}}}^{(1)}({{\bf{x}}}_{j})={[0,{x}_{j}^{3},0]}^{T}$ and ${{\bf{h}}}^{(2)}({{\bf{x}}}_{j},{{\bf{x}}}_{k})={[0,{x}_{j}^{2}{x}_{k},0]}^{T}$; ${{\bf{h}}}^{(1)}({{\bf{x}}}_{j})={[0,0,{x}_{j}^{3}]}^{T}$ and ${{\bf{h}}}^{(2)}({{\bf{x}}}_{j},{{\bf{x}}}_{k})={[0,0,{x}_{j}^{2}{x}_{k}]}^{T}$; ${{\bf{h}}}^{(1)}({{\bf{x}}}_{j})={[{y}_{j}^{3},0,0]}^{T}$ and ${{\bf{h}}}^{(2)}({{\bf{x}}}_{j}, {{\bf{x}}}_{k})= {[{y}_{j}^{2}{y}_{k},0,0]}^{T}$... ${{\bf{h}}}^{(1)}({{\bf{x}}}_{j})={[0,0,{z}_{j}^{3}]}^{T}$ and ${{\bf{h}}}^{(2)}({{\bf{x}}}_{j},{{\bf{x}}}_{k})={[0,0,{z}_{j}^{2}{z}_{k}]}^{T}$.

**Fig. 4:**
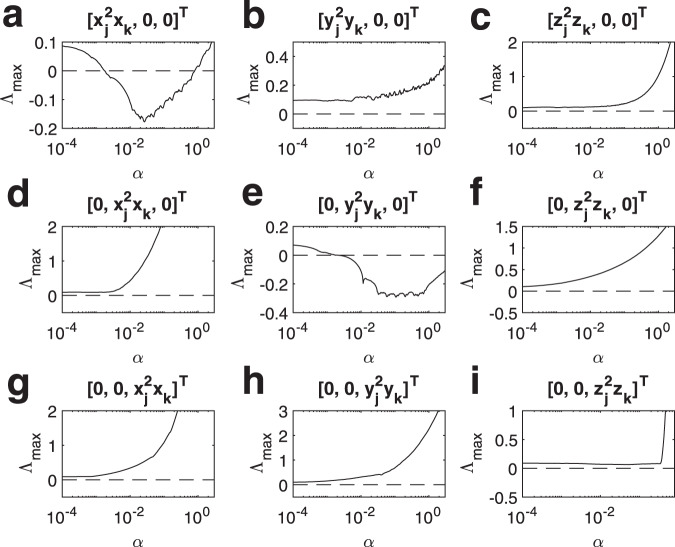
Synchronization in simplicial complexes of Rössler oscillators, in the case of natural coupling. The Master Stability Function is here calculated taking into account several coupling functions. **a** ${{\bf{h}}}^{(1)}({{\bf{x}}}_{j})={[{x}_{j}^{3},0,0]}^{T}$ and ${{\bf{h}}}^{(2)}({{\bf{x}}}_{j},{{\bf{x}}}_{k})={[{x}_{j}^{2}{x}_{k},0,0]}^{T}$, **b** ${{\bf{h}}}^{(1)}({{\bf{x}}}_{j})={[{y}_{j}^{3},0,0]}^{T}$ and ${{\bf{h}}}^{(2)}({{\bf{x}}}_{j},{{\bf{x}}}_{k})={[{y}_{j}^{2}{y}_{k},0,0]}^{T}$, **c** ${{\bf{h}}}^{(1)}({{\bf{x}}}_{j})={[{z}_{j}^{3},0,0]}^{T}$ and ${{\bf{h}}}^{(2)}({{\bf{x}}}_{j},{{\bf{x}}}_{k})={[{z}_{j}^{2}{z}_{k},0,0]}^{T}$, **d** ${{\bf{h}}}^{(1)}({{\bf{x}}}_{j})={[0,{x}_{j}^{3},0]}^{T}$ and ${{\bf{h}}}^{(2)}({{\bf{x}}}_{j},{{\bf{x}}}_{k})={[0,{x}_{j}^{2}{x}_{k},0]}^{T}$, **e** ${{\bf{h}}}^{(1)}({{\bf{x}}}_{j})={[0,{y}_{j}^{3},0]}^{T}$ and ${{\bf{h}}}^{(2)}({{\bf{x}}}_{j},{{\bf{x}}}_{k})={[0,{y}_{j}^{2}{y}_{k},0]}^{T}$, **f** ${{\bf{h}}}^{(1)}({{\bf{x}}}_{j})={[0,{z}_{j}^{3},0]}^{T}$ and ${{\bf{h}}}^{(2)}({{\bf{x}}}_{j},{{\bf{x}}}_{k})={[0,{z}_{j}^{2}{z}_{k},0]}^{T}$, **g** ${{\bf{h}}}^{(1)}({{\bf{x}}}_{j})={[0,0,{x}_{j}^{3}]}^{T}$ and ${{\bf{h}}}^{(2)}({{\bf{x}}}_{j},{{\bf{x}}}_{k})={[0,0,{x}_{j}^{2}{x}_{k}]}^{T}$, **h** ${{\bf{h}}}^{(1)}({{\bf{x}}}_{j})={[0,0,{y}_{j}^{3}]}^{T}$ and ${{\bf{h}}}^{(2)}({{\bf{x}}}_{j},{{\bf{x}}}_{k})={[0,0,{y}_{j}^{2}{y}_{k}]}^{T}$, **i** ${{\bf{h}}}^{(1)}({{\bf{x}}}_{j})={[0,0,{z}_{j}^{3}]}^{T}$ and ${{\bf{h}}}^{(2)}({{\bf{x}}}_{j},{{\bf{x}}}_{k})={[0,0,{z}_{j}^{2}{z}_{k}]}^{T}$.

**Fig. 5:**
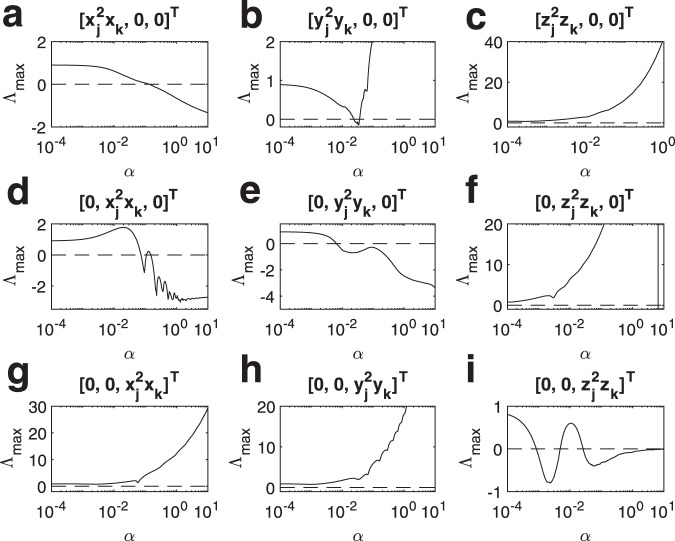
Synchronization in simplicial complexes of Lorenz systems, in the case of natural coupling. The Master Stability Function is here calculated taking into account several coupling functions. **a** ${{\bf{h}}}^{(1)}({{\bf{x}}}_{j})={[{x}_{j}^{3},0,0]}^{T}$ and ${{\bf{h}}}^{(2)}({{\bf{x}}}_{j},{{\bf{x}}}_{k})={[{x}_{j}^{2}{x}_{k},0,0]}^{T}$, **b** ${{\bf{h}}}^{(1)}({{\bf{x}}}_{j})={[{y}_{j}^{3},0,0]}^{T}$ and ${{\bf{h}}}^{(2)}({{\bf{x}}}_{j},{{\bf{x}}}_{k})={[{y}_{j}^{2}{y}_{k},0,0]}^{T}$, **c** ${{\bf{h}}}^{(1)}({{\bf{x}}}_{j})={[{z}_{j}^{3},0,0]}^{T}$ and ${{\bf{h}}}^{(2)}({{\bf{x}}}_{j},{{\bf{x}}}_{k})={[{z}_{j}^{2}{z}_{k},0,0]}^{T}$, **d** ${{\bf{h}}}^{(1)}({{\bf{x}}}_{j})={[0,{x}_{j}^{3},0]}^{T}$ and ${{\bf{h}}}^{(2)}({{\bf{x}}}_{j},{{\bf{x}}}_{k})={[0,{x}_{j}^{2}{x}_{k},0]}^{T}$, **e** ${{\bf{h}}}^{(1)}({{\bf{x}}}_{j})={[0,{y}_{j}^{3},0]}^{T}$ and ${{\bf{h}}}^{(2)}({{\bf{x}}}_{j},{{\bf{x}}}_{k})={[0,{y}_{j}^{2}{y}_{k},0]}^{T}$, **f** ${{\bf{h}}}^{(1)}({{\bf{x}}}_{j})={[0,{z}_{j}^{3},0]}^{T}$ and ${{\bf{h}}}^{(2)}({{\bf{x}}}_{j},{{\bf{x}}}_{k})={[0,{z}_{j}^{2}{z}_{k},0]}^{T}$, **g** ${{\bf{h}}}^{(1)}({{\bf{x}}}_{j})={[0,0,{x}_{j}^{3}]}^{T}$ and ${{\bf{h}}}^{(2)}({{\bf{x}}}_{j},{{\bf{x}}}_{k})={[0,0,{x}_{j}^{2}{x}_{k}]}^{T}$, **h** ${{\bf{h}}}^{(1)}({{\bf{x}}}_{j})={[0,0,{y}_{j}^{3}]}^{T}$ and ${{\bf{h}}}^{(2)}({{\bf{x}}}_{j},{{\bf{x}}}_{k})={[0,0,{y}_{j}^{2}{y}_{k}]}^{T}$, **i** ${{\bf{h}}}^{(1)}({{\bf{x}}}_{j})={[0,0,{z}_{j}^{3}]}^{T}$ and ${{\bf{h}}}^{(2)}({{\bf{x}}}_{j},{{\bf{x}}}_{k})={[0,0,{z}_{j}^{2}{z}_{k}]}^{T}$.

The results are shown in Fig. 4 for the Rössler oscillator and in Fig. 5 for the Lorenz system. Both cases exhibit a variety of behaviors that actually encompass all possible classes of MSF. In the case of Rössler oscillator we have one class III example (Fig. 4a), one class II example (Fig. 4e), while all remaining cases do correspond to class I. In the case of the Lorenz system we have several examples of class I behavior (Fig. 5c, f–h); three class II examples (Fig. 4a, d, e), and one class III example with a very narrow region for synchronization (Fig. 4b). Moreover, in Fig. 4i the MSF assumes negative values in two different intervals of *α*; overall, this represents a further example of class III behavior, providing however the extra scenario where increasing the coupling strength one can achieve alternating regions of synchronization and desynchronization.

### Real-world structures

As an example of a real-world structure, we apply our method to a social system modeling the interactions between the members of a university sport club, the so-called Zachary karate club data set^66^. The original social system is described in terms of a network consisting of *N* = 34 nodes and 78 links. Since the links form 45 triangles, several simplicial complexes can be constructed from this network, depending on which and how many nodes forming a triangle are effectively taken into consideration as being part of a 2-simplex or, on the contrary, as only connected by three pairwise interactions. In this way, we will be able to investigate the relevance of three-body interactions in mechanisms of collective behavior, such as consensus and synchronization, in social systems. It is indeed well known that pairwise interactions are not always enough to capture the complex behavior of many systems, including social systems^44^. For instance, processes of social contagion can occur in different ways, either through pairwise interactions (the links of a network), or in groups of three or more individuals (higher-order simplices), and it has been shown that models of diffusion on simplicial complexes can reproduce well the complex mechanisms of influence and reinforcement that are at work in the formation of opinions and in the adoption of novelties^67,68^. At first, let us consider the case where all triangles are considered as 2-simplexes. In this way, the members of the Zachary karate club may have both pairwise, when they are connected by a network link, and three-body interactions, when they belong to the same triangle (2-simplex). The presence of a link indicates a social interaction among the two nodes of the link, whereas a 2-simplex can be interpreted as a social interaction involving three members of the club, such as a discussion to which all of them simultaneously participated. Oscillators have usually been used to describe the units of a coupled dynamical system when modeling opinion formation in social systems^50,69^. As dynamical units we have decided to use chaotic oscillators, as it can be relevant to study synchronization in the more general scenario in which the opinions do not necessarily converge to a fixed stationary state^70,71^. In particular, we associate to each node a Rössler oscillator and focus on the class III case, selecting the coupling functions as ${{\bf{g}}}^{(1)}({{\bf{x}}}_{i},{{\bf{x}}}_{j})={[{x}_{j}^{3}-{x}_{i}^{3},0,0]}^{T}$ and ${{\bf{g}}}^{(2)}({{\bf{x}}}_{i},{{\bf{x}}}_{j},{{\bf{x}}}_{k})={[{x}_{j}^{2}{x}_{k}-{x}_{i}^{3},0,0]}^{T}$. With these assumptions, the dynamics of each node *i* is described by

21 $${\dot{x}}_{i}=	 \, -{y}_{i}-{z}_{i}+{\sigma }_{1}\mathop{\sum }\limits_{j=1}^{N}{a}_{ij}^{(1)}({x}_{j}^{3}-{x}_{i}^{3})\\ \, 	+{\sigma }_{2}\mathop{\sum }\limits_{j=1}^{N}\mathop{\sum }\limits_{k=1}^{N}{a}_{ijk}^{(2)}({x}_{j}^{2}{x}_{k}-{x}_{i}^{3}),\\ {\dot{y}}_{i}=	 \, {x}_{i}+a{y}_{i},\\ {\dot{z}}_{i}=	 \, b+{z}_{i}({x}_{i}-c).$$

**Fig. 6:**
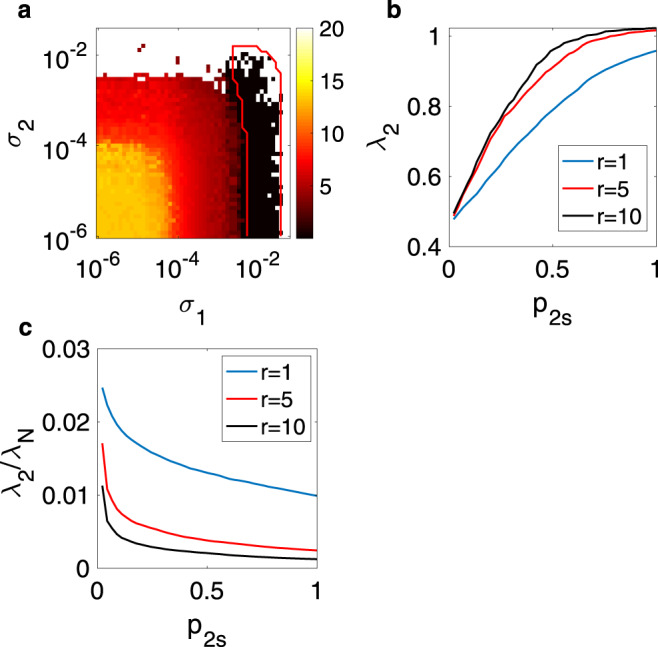
Synchronization in Zachary karate club structure. Synchronization is studied in simplicial complexes extracted from the interactions characterizing the Zachary karate club network. **a** Synchronization error (color code reported in the bar at the right of the panel) vs. *σ*_1_ and *σ*_2_ for the simplicial complex obtained when all the triangles are considered as being 2-simplexes. The red line delimits the area of stability of the synchronous solution predicted by the MSF. **b** *λ*_2_ vs. the percentage of 2-simplexes in the structure, *p*_2*s*_ (see text for definition); **c** *λ*_2_/*λ*_*N*_ vs. *p*_2*s*_. In **b** and **c** three different values of *r* are considered, with the color code for the plotted curves being reported in the corresponding insets.

Equations (21) are then simulated for different values of *σ*_1_ and *σ*_2_. The average synchronization error and the predictions provided by the MSF (15) are illustrated in Fig. 6a that shows the crucial role played by the pairwise links, as synchronization turns out to be impossible when only three-body interactions are considered, i.e., when *σ*_1_ = 0.

Next, we take the original network, and build different simplicial complexes by considering an increasing percentage (labeled as *p*_2*s*_) of triangles in the original structure as true 2-simplexes, that is, an increasing percentage of social interactions taking place among groups of three members of the club. For each of these structures, we determine the effective matrix ${\mathcal{M}}$ in (41), and calculate its spectrum of eigenvalues, and in particular we calculate the quantities ${\lambda }_{2}({\mathcal{M}})$ and ${\lambda }_{2}({\mathcal{M}})/{\lambda }_{N}({\mathcal{M}})$ (to simplify the notation here we shortly refer to these quantities as *λ*_2_ and *λ*_2_/*λ*_*N*_). The former quantity provides the scaling of synchronization for class II systems, while the latter quantity (*λ*_2_/*λ*_*N*_) is a proxy of synchronizability for class III systems. The larger are the two quantities, the easier is to obtain synchronization. Fig. 6b, c illustrates the results at three values of $r=\frac{{\sigma }_{2}}{{\sigma }_{1}}$. One finds that increasing *p*_2*s*_ has the effect of increasing *λ*_2_ (thus it facilitates synchronization in class II systems), but simultaneously dwindles *λ*_2_/*λ*_*N*_ (thus hindering synchronization in class III). Furthermore, Fig. 6 reveals that a larger value of $r=\frac{{\sigma }_{2}}{{\sigma }_{1}}$ leads to larger values of *λ*_2_, but smaller values of *λ*_2_/*λ*_*N*_, thus suggesting a beneficial impact of stronger three-body interactions for class II systems and an opposite effect on class III systems.

### Cluster synchronization in simplicial complexes

In complex networks, symmetries may induce cluster synchronization, a regime where nodes group into clusters of units synchronized to each other^30^. Network symmetries are permutations of the nodes preserving the connectivity pattern; they form a mathematical group, where each element may be represented by a permutation matrix R with elements *r*_*ij*_ = 1 if nodes *i* and *j* permute, and *r*_*ij*_ = 0 otherwise. The relevant property is that the orbits of the symmetry group associated to the network represent a partition into clusters that contain nodes that may synchronize each other. Group-theoretical considerations determine the exact composition and stability of clusters defined by the symmetry group^30^.

Extending the notion of cluster synchronization to simplicial complexes requires a formal definition of symmetries in the general framework of multi-body interactions. Although this goes beyond the purpose of this paper, here, we illustrate an example of a simplicial complex displaying cluster synchronization. The core idea is that symmetry-related nodes must be flow invariant. Since the flow now comprises pairwise as well as higher-order interactions, symmetries must preserve the invariance for all the interactions taking place in the simplicial complex. Note that the same general principle is at the basis of the onset of cluster synchronization in multilayer networks where the symmetries guarantee that synchronized nodes have equal dynamical variables when inter-layer coupling is also included^72^.

In simplicial complexes, the flow invariance of symmetry-related nodes is obtained when the same symmetries hold for all the Laplacians involved in the dynamical equations of the nodes. Indicating with R_*i*_ with *i* = 1, …, *n*_p_ the *n*_*p*_ permutation matrices describing representing the symmetry group associated to the cluster synchronization state, this requires that the Laplacians satisfy the following Lyapunov equations^30,73^:

22 $${{\rm{R}}}_{i}{{\mathcal{L}}}^{(1)}={{\mathcal{L}}}^{(1)}{{\rm{R}}}_{i}$$

and

23 $${{\rm{R}}}_{i}{{\mathcal{L}}}^{(2)}={{\mathcal{L}}}^{(2)}{{\rm{R}}}_{i}$$

for *i* = 1, …, *n*_*g*_.

**Fig. 7:**
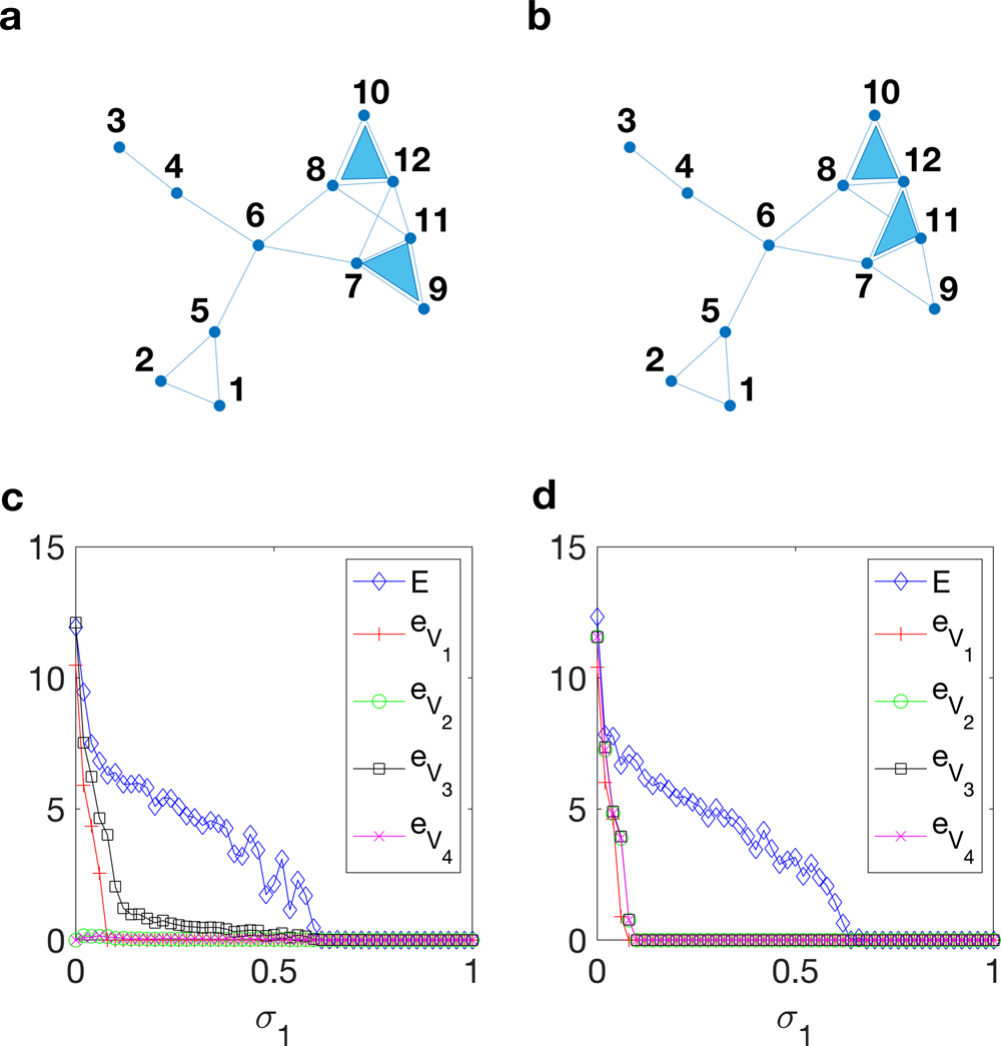
Cluster synchronization in simplicial complexes of Rössler oscillators. **a** A simplicial complex where the symmetries of ${{\mathcal{L}}}^{(2)}$ do not match those of ${{\mathcal{L}}}^{(1)}$. **b** A simplicial complex where the symmetries of ${{\mathcal{L}}}^{(2)}$ match those of ${{\mathcal{L}}}^{(1)}$. **c** Synchronization error as a function of *σ*_1_ for the simplicial complex ia. **d** Synchronization error as a function of *σ*_1_ for the simplicial complex in panel b. In both cases, *σ*_2_ has been set to *σ*_2_ = 0.2.

To illustrate our results, we consider the two simplicial complexes shown in Fig. 7a, b, that have the same set of links, but different 2-simplices. The symmetries existing for the common network backbone induce the following partition of the nodes: *V*_1_ = {1, 2}, *V*_2_ = {7, 8}, *V*_3_ = {9, 10}, *V*_4_ = {11, 12}, *V*_5_ = {3}, *V*_6_ = {4}, *V*_7_ = {5}, *V*_8_ = {6}. Hence, the two simplicial complexes satisfy Eq. (22) for this symmetry group. However, for the simplicial complex in Fig. 7a, Eq. (22) do not hold, while the simplicial complex in Fig. 7b satisfies them.

We numerically study cluster synchronization monitoring the average synchronization error in each non-trivial cluster ${e}_{{V}_{h}}={\left\langle \sqrt{{\sum }_{i,j\in {V}_{h}}\parallel {{\bf{x}}}_{i}(t)-{{\bf{x}}}_{j}(t){\parallel }^{2}}\right\rangle }_{T}$ for *h* = 1, …, 4 and the average overall synchronization error *E* as in Eq. (45) to measure the onset of complete synchronization. Figure 7c, d shows the results for *σ*_1_ ∈ [0, 1] and *σ*_2_ = 0.2. As it can be observed, for the simplicial complex of Fig. 7b there is a interval of values of *σ*_1_, i.e., *σ*_1_ ∈ [0.1, 0.62], where the units in the four clusters are synchronized in the absence of complete synchronization, whereas for the simplicial complex of Fig. 7a *V*_1_ is the only cluster that synchronizes before complete synchronization is achieved. These results show that higher-order interactions can modulate the pattern of synchronization emerging in the simplicial complex, as a diverse arrangement of the same number of 2-simplices (two in our example) led to different synchronous clusters.

## Discussion

Collective emergent phenomena in complex systems are the result of the interactions of many elementary systems, that may occur through different mechanisms. We have here formulated the most general model accounting for many-body interactions of arbitrary order among dynamical systems of arbitrary nature, and we have given explicit necessary conditions for synchronization to set up in these structures in a stable way.

Under the only hypothesis of non-invasiveness of the coupling functions (which is the only assumption impossible to be disregarded, as it is the fundamental basis for the very same existence and invariance of the synchronization solution), we have derived the conditions for stability of the synchronous motion, which involve the use of generalized Laplacian matrices mapping the effects of high-order interactions. Moreover, we have even shown that, in some relevant cases, our approach ultimately provides a MSF, which formalizes the interplay between topology of the simplicial complex and dynamics of the single units. Finally, our theoretical derivations have been complemented by a series of numerical results, which have fully confirmed the validity and generality of the approach, and case studies, where our technique crucially enables to take into account the fundamental presence of higher-order interactions, whose effect, previously, was not possible to address.

We note that our method is based on linear stability, therefore providing a local analysis of synchronization. This analysis can be complemented by other techniques, such as the basin stability^74^, aiming at characterizing the basin of attraction of the synchronous manifold. Similar techniques require extensive numerical simulations of the full nonlinear model for many different initial conditions, but provide an important characterization of the system behavior, specially in the presence of multistability.

Our results pave the way to several novel studies. First, the generality of the assumptions made renders it applicable in a wide range of practical cases, and we expect that our method could be of value in a plethora of experimental and/or practical circumstances, in order to make a series of a-priori predictions on the emergence of synchronization.

Second, the fact that our method can be used irrespectively on the coupling functions offers the possibility to apply it for the investigation of diverse coupling mechanisms that may occur at different orders of the interactions. In particular, questions like what exact role do such interactions play in shaping the path to synchronization and its robustness against heterogeneities in the oscillator dynamics, or what is the difference in using one or another coupling mechanism, can actually be tackled and clarified by our approach. Answering these questions, indeed, is of crucial importance from the perspective of engineering mechanisms for achieving synchronization in man-made systems. For instance, power grids are currently synchronized by exploiting only pairwise interactions, whereas more functional and more performing configurations could be designed, thanks to our method, by the use of higher order interactions.

Third, our study focuses on what is possibly the most common and widely studied form of synchronization, that is, the regime where all the units follow the same trajectory. However, as also mentioned in the introduction, many other different forms of synchronization exist, including cluster synchronization, Chimera and Bellerophon states, remote synchronization, etc... All such states have been so far studied in structures with pairwise interactions. The emergence of such states, or even of novel ones, in simplicial complexes, as well as their stability, are very intriguing problems and certainly constitute directions for further research (an example limited to the case of cluster synchronization has been discussed in “Results”).

## Methods

### Networks and higher-order structures of interactions

A network is a collection of nodes and of edges connecting pairs of nodes. Mathematically, it is represented by a graph ${\mathcal{G}}=({\mathcal{V}},{\mathcal{E}})$, which consists of a set ${\mathcal{V}}$ with $N=| {\mathcal{V}}|$ elements called vertices (or nodes), and a set ${\mathcal{E}}$ whose *K* elements, called edges or links, are pairs of nodes (*i*, *j*) (*i*, *j* = 1, 2, …, *N* and *i* ≠ *j*). As graphs explicitly refer to pairwise interactions, networks have been very successful in capturing the properties of coupled dynamical systems in all such cases in which the interactions can be expressed (or approximated) as a sum of two-body terms^75^. Conversely, their limits emerge when it comes to model higher-order interactions. In fact, the presence of a triangle of three nodes *i*, *j*, *k* in a network, e.g., the presence of the three links (*i*, *j*), (*i*, *k*), (*j*, *k*) in the corresponding graph, is not able to capture the difference between a three-body interaction of the three individuals, from the sum of three pairwise interactions. Notice that these are two completely different situations, with completely different social mechanisms and dynamics at work^67^.

Simplicial complexes are instead the proper mathematical structures for describing high-order interactions. A simplicial complex is an aggregate of simplices, objects that generalize links and can in general be of different dimension. A *d*-simplex, or simplex of dimension *d*, *σ* is, in its simplest definition, a collection of *d* + 1 nodes. In this way, a 0-simplex is a node, a 1-simplex is a link, a 2-simplex (*i*, *j*, *k*) is a two-dimensional object made by three nodes, usually called a (full) triangle, a 3-simplex is a tetrahedron, i.e., a three-dimensional object and so on. It is now possible to differentiate between a three-body interaction, and three bodies in pairwise interactions: the first case will be represented by a complete triangle, a two-dimensional simplex, while the second case will consist of three one-dimensional objects. Hence, in the following of this paper, simplices of dimension *d* will be used to describe the structure of (*d* + 1)-body interactions.

Finally, a simplicial complex ${\mathcal{S}}$ on a given set of nodes ${\mathcal{V}}$, with $| {\mathcal{V}}| =N$, is a collection of *M* simplices, ${\mathcal{S}}=\{{\sigma }_{1},{\sigma }_{2},\ldots ,{\sigma }_{M}\}$, with the extra requirement that, for any simplex $\sigma \in {\mathcal{S}}$, all the simplices $\sigma ^{\prime}$ with $\sigma ^{\prime} \subset \sigma$, i.e., all the simplices built from subsets of *σ*, are also contained in ${\mathcal{S}}$. Due to this requirement, simplicial complexes are a very particular type of hypergraphs^76^. Simplicial complexes have shown to be appropriate in the context of social systems^67,77,78^ and they will turn very useful to study coupled dynamical systems. We indicate as *M*_*d*_, *d* = 1, 2, …, *D* the number of *d*-simplices present in ${\mathcal{S}}$ (where *D*, the order of the simplicial complex, is the dimension of the largest simplex in ${\mathcal{S}}$), with the constraint that $\mathop{\sum }\nolimits_{d=1}^{D}{M}_{d}=M$.

As a mathematical representation of simplicial complexes, we use here a formalism which generalizes directly the concept of adjacency matrix for a network. The adjacency matrix A of a graph ${\mathcal{G}}$ is a *N* × *N* matrix, such that entry *a*_*ij*_ is 1 when edge $(i,j)\in {\mathcal{E}}$, and 0 otherwise. The idea can be extended to simplicial complexes by considering tensors instead of matrices. In fact, for each dimension *d*, we can define the ${\underbrace {N \times N \times \ldots \times N}}_{d + 1}$ adjacency tensor A^(*d*)^, whose entry ${a}_{{i}_{1},\ldots ,{i}_{d+1}}^{(d)}$ is equal to 1 if the *d*-simplex (*i*_1_, …, *i*_*d*+1_) belongs to the simplex ${\mathcal{S}}$, and is 0 otherwise^17^. Notice that each tensor is symmetric with respect to its *d* + 1 indices, which means that the value of a given entry ${a}_{{i}_{1},\ldots ,{i}_{d+1}}^{(d)}$ is equal to the value of the entries corresponding to any permutation of the indices.

With the definition above, A^(1)^ coincides with the standard adjacency matrix A, while the *N*  ×  *N*  ×  *N* adjacency tensor A^(2)^ characterizes two-dimensional objects: one has ${a}_{ijk}^{(2)}=1$ if the three nodes *i*, *j*, *k* form a full triangle, and otherwise ${a}_{ijk}^{(2)}=0$. As a conclusion, it is possible to map completely the connectivity structure of a simplicial complex ${\mathcal{S}}$ into the entire set of *D* adjacency tensors A^(*d*)^, *d* = 1, 2, …*, D*.

A node *i* of a simplicial complex ${\mathcal{S}}$ cannot be, therefore, characterized only by giving its degree ${k}_{i}=\mathop{\sum }\nolimits_{j=1}^{N}{a}_{ij}^{(1)}$, but one needs instead to account for the number of simplices of any dimension, incident in *i*. It is therefore extremely useful to define the generalized d-degree, ${k}_{i}^{(d)}$, of a node *i* as

24 $${k}_{i}^{(d)}=\frac{1}{d!}\mathop{\sum }\limits_{{i}_{1}=1}^{N}\mathop{\sum }\limits_{{i}_{2}=1}^{N}\ldots \mathop{\sum }\limits_{{i}_{d}=1}^{N}{a}_{i,{i}_{1},{i}_{2},\ldots ,\,{i}_{d}}^{(d)},$$

with *d* = 1, 2, …, *D* so that ${k}_{i}^{(1)}$ coincides with the standard degree of node *i*, ${k}_{i}^{(2)}$ counts the number of triangles (2-simplices) to which *i* participates

25 $${k}_{i}^{(2)}=1/2\mathop{\sum }\limits_{j=1}^{N}\mathop{\sum }\limits_{k=1}^{N}{a}_{ijk}^{(2)},$$

${k}_{i}^{(3)}$ the number of tetrahedrons, and so on.

Analogously, we can also define the generalized d-degree ${k}_{ij}^{(d)}$ of a link (*i*, *j*) as the number of *d*-simplices to which link (*i*, *j*) is part of. We can write its expression in terms of the adjacency tensor A^(*d*)^ of dimension *d*, with *d* = 1, 2, …, *D*, as^17^

26 $${k}_{ij}^{(d)}=\frac{1}{(d-1)!}\mathop{\sum }\limits_{{i}_{1}=1}^{N}\mathop{\sum }\limits_{{i}_{2}=1}^{N}\ldots \mathop{\sum }\limits_{{i}_{d-1}=1}^{N}{a}_{i,j,{i}_{1},{i}_{2},\ldots ,{i}_{d-1}}^{(d)},$$

so that ${k}_{ij}^{(1)}={a}_{ij}^{(1)}$, while ${k}_{ij}^{(2)}$ counts the number of triangles (2-simplices) to which (*i*, *j*) participates

27 $${k}_{ij}^{(2)}=\mathop{\sum }\limits_{k=1}^{N}{a}_{ijk}^{(2)},$$

and so on.

The Laplacian is a matrix that is of particular importance in many linear processes such as diffusion in graphs, but also turns useful in the linearization of nonlinear systems, for instance when we study the stability of a synchronized state in a networked dynamical system. The Laplacian matrix *L* = {*l*_*ij*_} of a graph can be defined as *L* = *K* − *A*, where *K* is the diagonal matrix having the node degrees as diagonal elements. We give here a definition of generalized Laplacian describing the case of systems with high-order interactions. The generalized Laplacian of order *d*, with *d* = 1, 2, …, *D*, is a matrix ${{\mathcal{L}}}^{(d)}$ whose elements are defined as

28 $${{\mathcal{L}}}_{ij}^{(d)}=\left\{\begin{array}{lll}0&\,{\text{for}}\,& i\ne j \quad\,{\text{and}}\,\quad {a}_{ij}^{(1)}=0\\ -(d-1)!{k}_{ij}^{(d)}&\,{\text{for}}\,& i\ne j\quad\,{\text{and}}\,\quad {a}_{ij}^{(1)}=1\\ d!{k}_{i}^{(d)}&\,{\text{for}}\,& i=j,\hfill\end{array}\right.$$

where ${k}_{ij}^{(d)}$ is the generalized *d*-degree of the link (*i*, *j*), and ${k}_{i}^{(d)}$ is the generalized *d*-degree of node *i*. Replacing Eqs. (24) and (26) in Eq. (28), in the case *D* = 2, we get an equivalent expression for the generalized Laplacian:

29 $${{\mathcal{L}}}_{ij}^{(2)}=\left\{\begin{array}{ll}-\sum _{k}{a}_{ijk}^{(2)}&\,{\text{for}}\hfill\,\quad i\ne j\quad\\ -\sum _{\ell \ne i}{{\mathcal{L}}}_{i\ell }^{(2)}&\,{\text{for}}\,\qquad i=j,\end{array}\right.$$

Notice that ${{\mathcal{L}}}^{(1)}$ recovers exactly the classical Laplacian matrix. This definition of generalized Laplacian will turn useful in our study.

### Derivation of Eq. (3)

To derive the conditions for the stability of the synchronization solution **x**^*s*^, one first considers small perturbations around the synchronous state, i.e., *δ***x**_*i*_ = **x**_*i*_ − **x**^*s*^, and performs a linear stability analysis of Eq. (2). One has

30 $${\dot{\delta {\bf{x}}}}_{i}=	 \, J{\bf{f}}({{\bf{x}}}^{s})\delta {{\bf{x}}}_{i}+{\sigma }_{1}\mathop{\sum }\limits_{j=1}^{N}{a}_{ij}^{(1)}\left[\frac{\partial {{\bf{g}}}^{(1)}({{\bf{x}}}_{i},{{\bf{x}}}_{j})}{\partial {{\bf{x}}}_{i}}{| }_{({{\bf{x}}}^{s},{{\bf{x}}}^{s})}\delta {{\bf{x}}}_{i}\right. \left.+\frac{\partial {{\bf{g}}}^{(1)}({{\bf{x}}}_{i},{{\bf{x}}}_{j})}{\partial {{\bf{x}}}_{j}}{| }_{({{\bf{x}}}^{s},{{\bf{x}}}^{s})}\delta {{\bf{x}}}_{j}\right]\\ \, 	+{\sigma }_{2}\mathop{\sum }\limits_{j=1}^{N}\mathop{\sum }\limits_{k=1}^{N}{a}_{ijk}^{(2)}\left[\frac{\partial {{\bf{g}}}^{(2)}({{\bf{x}}}_{i},{{\bf{x}}}_{j},{{\bf{x}}}_{k})}{\partial {{\bf{x}}}_{i}}{| }_{({{\bf{x}}}^{s},{{\bf{x}}}^{s},{{\bf{x}}}^{s})}\delta {{\bf{x}}}_{i}\right. +\frac{\partial {{\bf{g}}}^{(2)}({{\bf{x}}}_{i},{{\bf{x}}}_{j},{{\bf{x}}}_{k})}{\partial {{\bf{x}}}_{j}}{| }_{({{\bf{x}}}^{s},{{\bf{x}}}^{s},{{\bf{x}}}^{s})}\delta {{\bf{x}}}_{j}\\ \, 	\left.+\frac{\partial {{\bf{g}}}^{(2)}({{\bf{x}}}_{i},{{\bf{x}}}_{j},{{\bf{x}}}_{k})}{\partial {{\bf{x}}}_{k}}{| }_{({{\bf{x}}}^{s},{{\bf{x}}}^{s},{{\bf{x}}}^{s})}\delta {{\bf{x}}}_{k}\right],$$

where *J***f**(**x**^*s*^) denotes the *m* × *m* Jacobian matrix of the function **f**, evaluated at the synchronous state **x**^*s*^. The first, very important, conceptual step in our derivation consists in noticing that all coupling functions are synchronization noninvasive (i.e., **g**^(1)^(**x**, **x**) ≡ 0 and **g**^(2)^(**x**, **x**, **x**) ≡ 0). As their value is then constant (equal to zero) at the synchronization manifold, it immediately follows that their total derivative vanishes as well, which implies on its turn that

31 $$	\,\frac{\partial {{\bf{g}}}^{(1)}({{\bf{x}}}_{i},{{\bf{x}}}_{j})}{\partial {{\bf{x}}}_{i}}{\Bigg| }_{({{\bf{x}}}^{s},{{\bf{x}}}^{s})}+\frac{\partial {{\bf{g}}}^{(1)}({{\bf{x}}}_{i},{{\bf{x}}}_{j})}{\partial {{\bf{x}}}_{j}}{\Bigg| }_{({{\bf{x}}}^{s},{{\bf{x}}}^{s})}= 0,\\ 	\,\frac{\partial {{\bf{g}}}^{(2)}({{\bf{x}}}_{i},{{\bf{x}}}_{j},{{\bf{x}}}_{k})}{\partial {{\bf{x}}}_{i}}{\Bigg| }_{({{\bf{x}}}^{s},{{\bf{x}}}^{s},{{\bf{x}}}^{s})}+\frac{\partial {{\bf{g}}}^{(2)}({{\bf{x}}}_{i},{{\bf{x}}}_{j},{{\bf{x}}}_{k})}{\partial {{\bf{x}}}_{j}}{\Bigg| }_{({{\bf{x}}}^{s},{{\bf{x}}}^{s},{{\bf{x}}}^{s})}+ \\ \, 	\quad+\frac{\partial {{\bf{g}}}^{(2)}({{\bf{x}}}_{i},{{\bf{x}}}_{j},{{\bf{x}}}_{k})}{\partial {{\bf{x}}}_{k}}{\Bigg| }_{({{\bf{x}}}^{s},{{\bf{x}}}^{s},{{\bf{x}}}^{s})}= 0.$$

Then, one can factor out the terms $\frac{\partial {{\bf{g}}}^{(1)}({{\bf{x}}}_{i},{{\bf{x}}}_{j})}{\partial {{\bf{x}}}_{i}}{| }_{({{\bf{x}}}^{s},{{\bf{x}}}^{s})}\delta {{\bf{x}}}_{i}$ and $\frac{\partial {{\bf{g}}}^{(2)}({{\bf{x}}}_{i},{{\bf{x}}}_{j},{{\bf{x}}}_{k})}{\partial {{\bf{x}}}_{i}}{| }_{({{\bf{x}}}^{s},{{\bf{x}}}^{s},{{\bf{x}}}^{s})}\delta {{\bf{x}}}_{i}$ in the summations (both of them, indeed, do not depend on the indices of the summations). Furthermore, one has that $\mathop{\sum }\nolimits_{j=1}^{N}{a}_{ij}^{(1)}={k}_{i}^{(1)}$ and $\mathop{\sum }\limits_{j=1}^{N}\mathop{\sum }\nolimits_{k=1}^{N}{a}_{ijk}^{(2)}=2{k}_{i}^{(2)}$. Plugging back the resulting terms inside the summations, and using Eq. (31), one eventually obtains

32 $${\dot{\delta {\bf{x}}}}_{i}=	 \, J{\bf{f}}({{\bf{x}}}^{s})\delta {{\bf{x}}}_{i}-{\sigma }_{1}\mathop{\sum }\limits_{j=1}^{N}{{\mathcal{L}}}_{ij}^{(1)}J{{\bf{g}}}^{(1)}({{\bf{x}}}^{s},{{\bf{x}}}^{s})\delta {{\bf{x}}}_{j}\\ \, 	-{\sigma }_{2}\mathop{\sum }\limits_{j=1}^{N}\mathop{\sum }\limits_{k=1}^{N}{\tau }_{ijk}\left[{J}_{1}{{\bf{g}}}^{(2)}({{\bf{x}}}^{s},{{\bf{x}}}^{s},{{\bf{x}}}^{s})\delta {{\bf{x}}}_{j} +{J}_{2}{{\bf{g}}}^{(2)}({{\bf{x}}}^{s},{{\bf{x}}}^{s},{{\bf{x}}}^{s})\delta {{\bf{x}}}_{k}\right],$$

where we introduced a tensor T whose elements are ${\tau }_{ijk}=2{k}_{i}^{(2)}{\delta }_{ijk}-{a}_{ijk}^{(2)}$ for *i*, *j*, *k* = 1, …, *N*, and simplified the notation as

33 $$J{{\bf{g}}}^{(1)}({{\bf{x}}}^{s},{{\bf{x}}}^{s})	= \, \frac{\partial {{\bf{g}}}^{(1)}({{\bf{x}}}_{i},{{\bf{x}}}_{j})}{\partial {{\bf{x}}}_{j}}{| }_{({{\bf{x}}}^{s},{{\bf{x}}}^{s})},\\ {J}_{1}{{\bf{g}}}^{(2)}({{\bf{x}}}^{s},{{\bf{x}}}^{s},{{\bf{x}}}^{s})	= \, \frac{\partial {{\bf{g}}}^{(2)}({{\bf{x}}}_{i},{{\bf{x}}}_{j},{{\bf{x}}}_{k})}{\partial {{\bf{x}}}_{j}}{| }_{({{\bf{x}}}^{s},{{\bf{x}}}^{s},{{\bf{x}}}^{s})},\\ {J}_{2}{{\bf{g}}}^{(2)}({{\bf{x}}}^{s},{{\bf{x}}}^{s},{{\bf{x}}}^{s})	= \, \frac{\partial {{\bf{g}}}^{(2)}({{\bf{x}}}_{i},{{\bf{x}}}_{j},{{\bf{x}}}_{k})}{\partial {{\bf{x}}}_{k}}{| }_{({{\bf{x}}}^{s},{{\bf{x}}}^{s},{{\bf{x}}}^{s})}.$$

Already at this stage, it is fundamental to remark that our approach does not require a diffusive functional form for the interplay among the network nodes, and therefore we are actually encompassing an extremely broad class of coupling functions. For instance, our approach allows the formal treatment of the Kuramoto model^50^, where *m* = 1, each network unit *i* is identified by the instantaneous phase *θ*_*i*_ of an oscillator, and the coupling between nodes *i* and *j* is given by the function $\sin ({\theta }_{j}-{\theta }_{i})$, which is not diffusive.

Let us now make our second, conceptual, step, which will allow us to greatly simplify the last term on the right hand side of Eq. (32). Such a term refers to three-body interactions, and we now show how to map it into a single summation involving the generalized Laplacian matrix. This is done by remarking that the two Jacobian matrices *J*_1_**g**^(2)^(**x**^*s*^, **x**^*s*^, **x**^*s*^) and *J*_2_**g**^(2)^(**x**^*s*^, **x**^*s*^, **x**^*s*^) are both independent on *k* and *j*. Accordingly, Eq. (32) becomes

34 $${\dot{\delta}} {\bf{x}}_{i}=	 \, J{\bf{f}}({{\bf{x}}}^{s})\delta {{\bf{x}}}_{i}-{\sigma }_{1}\mathop{\sum }\limits_{j=1}^{N}{{\mathcal{L}}}_{ij}^{(1)}J{{\bf{g}}}^{(1)}({{\bf{x}}}^{s},{{\bf{x}}}^{s})\delta {{\bf{x}}}_{j}\\ \, 	-{\sigma }_{2}\left[\sum_{j=1}^{N}{J}_{1}{{\bf{g}}}^{(2)}({{\bf{x}}}^{s},{{\bf{x}}}^{s},{{\bf{x}}}^{s})\delta {{\bf{x}}}_{j}\sum_{k=1}^{N}{\tau }_{ijk} +\sum_{k=1}^{N}{J}_{2}{{\bf{g}}}^{(2)}({{\bf{x}}}^{s},{{\bf{x}}}^{s},{{\bf{x}}}^{s})\delta {{\bf{x}}}_{k}\sum_{j=1}^{N}{\tau }_{ijk}\right].$$

Then, using the symmetric property of T, namely $\sum _{k}{\tau }_{ijk}=\sum _{k}{\tau }_{ikj}$, we have

35 $${\dot{\delta {\bf{x}}}}_{i}=	 \, J{\bf{f}}({{\bf{x}}}^{s})\delta {{\bf{x}}}_{i}-{\sigma }_{1}\mathop{\sum }\limits_{j=1}^{N}{{\mathcal{L}}}_{ij}^{(1)}J{{\bf{g}}}^{(1)}({{\bf{x}}}^{s},{{\bf{x}}}^{s})\delta {{\bf{x}}}_{j}\\ \, 	-{\sigma }_{2}\left[\mathop{\sum }\limits_{j=1}^{N}{J}_{1}{{\bf{g}}}^{(2)}({{\bf{x}}}^{s},{{\bf{x}}}^{s},{{\bf{x}}}^{s})\delta {{\bf{x}}}_{j}{{\mathcal{L}}}_{ij}^{(2)}\right. \left.+\mathop{\sum }\limits_{k=1}^{N}{J}_{2}{{\bf{g}}}^{(2)}({{\bf{x}}}^{s},{{\bf{x}}}^{s},{{\bf{x}}}^{s})\delta {{\bf{x}}}_{k}{{\mathcal{L}}}_{ik}^{(2)}\right]\\ =	 \, J{\bf{f}}({{\bf{x}}}^{s})\delta {{\bf{x}}}_{i}-{\sigma }_{1}\mathop{\sum }\limits_{j=1}^{N}{{\mathcal{L}}}_{ij}^{(1)}J{{\bf{g}}}^{(1)}({{\bf{x}}}^{s},{{\bf{x}}}^{s})\delta {{\bf{x}}}_{j} -{\sigma }_{2}\mathop{\sum }\limits_{j=1}^{N}{{\mathcal{L}}}_{ij}^{(2)}\left[{J}_{1}{{\bf{g}}}^{(2)}({{\bf{x}}}^{s},{{\bf{x}}}^{s},{{\bf{x}}}^{s})\right.\\ \, 	\left.+{J}_{2}{{\bf{g}}}^{(2)}({{\bf{x}}}^{s},{{\bf{x}}}^{s},{{\bf{x}}}^{s})\right]\delta {{\bf{x}}}_{j}.$$

Let us now rewrite Eq. (35) in block form by introducing the stack vector $\delta {\bf{x}}={[\delta {{\bf{x}}}_{1}^{T},\delta {{\bf{x}}}_{2}^{T},\ldots ,\delta {{\bf{x}}}_{N}^{T}]}^{T}$ and denoting by JF = *J***f**(**x**^*s*^), JG^(1)^ = *J***g**^(1)^(**x**^*s*^, **x**^*s*^) and JG^(2)^ = *J*_1_**g**^(2)^(**x**^*s*^, **x**^*s*^, **x**^*s*^) + *J*_2_**g**^(2)^(**x**^*s*^, **x**^*s*^, **x**^*s*^). One obtains

36 $$\dot{\delta {\bf{x}}}=\left[{{\rm{I}}}_{N}\otimes {\rm{JF}}-{\sigma }_{1}{{\mathcal{L}}}^{(1)}\otimes {{\rm{JG}}}^{(1)}-{\sigma }_{2}{{\mathcal{L}}}^{(2)}\otimes {{\rm{JG}}}^{(2)}\right]\delta {\bf{x}}.$$

The third, and final, conceptual step is to remark that all generalized Laplacians ${{\mathcal{L}}}^{(d)}$ are symmetric real-valued zero-row-sum matrices. Therefore: (i) they are all diagonalizable; (ii) for each one of them the set of eigenvalues is made of real non-negative numbers, and the corresponding set of eigenvectors constitutes a orthonormal basis of ${{\mathbb{R}}}^{N}$; (iii) they all share, as the smallest of their eigenvalues, *λ*_1_ ≡ 0, whose associated eigenvector $\frac{1}{\sqrt{N}}\,{(1,1,1,...,1)}^{T}$ is aligned along the synchronization manifold; (iv) as in general they do not commute, the sets of eigenvectors corresponding to all others of their eigenvalues are different from one another, and yet any perturbation to the synchronization manifold (which, by definition, lies in the tangent space) can be expanded as linear combination of one whatever of such eigenvector sets (the relevant consequence is that one can arbitrarily select any of the generalized Laplacians as the reference for the choice of the basis of the transverse space, and all other eigenvector sets will map to such a basis by means of unitary matrix transformations).

We are then fully entitled to take, as reference basis, the one constituted by the eigenvectors of the classic Laplacian ${{\mathcal{L}}}^{(1)}$ (V = [**v**_1_, **v**_2_, …, **v**_*N*_]), and consider new variables *η* = (V^−1^ ⊗ I_*m*_)**δx**. We get

37 $$\dot{{\eta }}=	 \, ({{\rm{V}}}^{-1}\otimes {{\rm{I}}}_{m})\left[{{\rm{I}}}_{N}\otimes {\rm{JF}}-{\sigma }_{1}{{\mathcal{L}}}^{(1)}\otimes {{\rm{JG}}}^{(1)}\right.\\ 	\,\,\left.-{\sigma }_{2}{{\mathcal{L}}}^{(2)}\otimes {{\rm{JG}}}^{(2)}\right]({\rm{V}}\otimes {{\rm{I}}}_{m}){\eta }.$$

Furthermore, taking into account that ${{\rm{V}}}^{-1}{{\mathcal{L}}}^{(1)}{\rm{V}}={\rm{diag}}({\lambda }_{1},{\lambda }_{2},\ldots ,{\lambda }_{N})={{\rm{{{\Lambda }}}}}^{(1)}$, where 0 = *λ*_1_ < *λ*_2_ ≤ …*λ*_*N*_ are the eigenvalues of ${{\mathcal{L}}}^{(1)}$, and indicating with ${{\tilde{{\mathcal{L}}}}^{(2)}={{\rm{V}}}^{-1}{{\mathcal{L}}}^{(2)}{\rm{V}}}$ the transformed generalized Laplacian of order 2, one obtains that

38 $$\dot{{\eta }}=\left[{{\rm{I}}}_{N}\otimes {\rm{JF}}-{\sigma }_{1}{{\rm{{{\Lambda }}}}}^{(1)}\otimes {{\rm{JG}}}^{(1)}-{\sigma }_{2}{\tilde{{\mathcal{L}}}}^{(2)}\otimes {{\rm{JG}}}^{(2)}\right]{\eta }.$$

As ${{\mathcal{L}}}^{(2)}$ is zero-row sum (i.e. ${{\mathcal{L}}}^{(2)}{{\bf{v}}}_{1}=0$), Eq. (3) are finally obtained from Eq. (38).

### Derivation of the MSF in the case of natural coupling

In the case of natural coupling as in Eq. (14), one has that *J*_1_**h**^(2)^(**x**^*s*^, **x**^*s*^) + *J*_2_**h**^(2)^(**x**^*s*^, **x**^*s*^) = *J***h**^(1)^(**x**^*s*^). The consequence is that the equations of the linearized dynamics in Eq. (35) can be rewritten as follows

39 $${\dot{\delta {\bf{x}}}}_{i}=	 \, J{\bf{f}}({{\bf{x}}}^{s})\delta {{\bf{x}}}_{i}-{\sigma }_{1}\mathop{\sum }\limits_{j=1}^{N}{{\mathcal{L}}}_{ij}^{(1)}J{{\bf{h}}}^{(1)}({{\bf{x}}}^{s})\delta {{\bf{x}}}_{j}\\ \, 	-{\sigma }_{2}\mathop{\sum }\limits_{j=1}^{N}{{\mathcal{L}}}_{ij}^{(2)}J{{\bf{h}}}^{(1)}({{\bf{x}}}^{s})\delta {{\bf{x}}}_{j}\\ =	 \, J{\bf{f}}({{\bf{x}}}^{s})\delta {{\bf{x}}}_{i}\\ \, 	-\mathop{\sum }\limits_{j=1}^{N}\left[{\sigma }_{1}{{\mathcal{L}}}_{ij}^{(1)}+{\sigma }_{2}{{\mathcal{L}}}_{ij}^{(2)}\right]J{{\bf{h}}}^{(1)}({{\bf{x}}}^{s})\delta {{\bf{x}}}_{j}.$$

Alternatively, one can consider the zero-row sum, symmetric, effective matrix ${\mathcal{M}}$, given by

40 $${\mathcal{M}}={{\mathcal{L}}}^{(1)}+r{{\mathcal{L}}}^{(2)},\qquad r=\frac{{\sigma }_{2}}{{\sigma }_{1}}.$$

and rewrite Eq. (39) as follows

41 $${\dot{\delta {\bf{x}}}}_{i}=J{\bf{f}}({{\bf{x}}}^{s})\delta {{\bf{x}}}_{i}-{\sigma }_{1}\mathop{\sum }\limits_{j=1}^{N}{{\mathcal{M}}}_{ij}\,J{{\bf{h}}}^{(1)}({{\bf{x}}}^{s})\delta {{\bf{x}}}_{j}.$$

where we notice that the eigenvalues of ${\mathcal{M}}$ depend on the ratio *r* of the coupling coefficients.

Equation (41) allows to establish a formal full analogy between the case of a simplicial complex and that of a network with weights given by the coefficients of the effective matrix ${\mathcal{M}}$. In this case, by diagonalizing the effective matrix ${\mathcal{M}}$, the transverse modes can be fully decoupled such that Eq. (15) is obtained, which prompts for the definition of a single-parameter MSF.

### Numerical simulations

In our numerical simulations, we used two paradigmatic chaotic systems for the study of synchronization in systems of coupled units. The isolated dynamics of the Rössler oscillator is described by

42 $$\dot{x}	= \, -y-z,\\ \dot{y}	= \, x+ay,\\ \dot{z}	= \, b+z(x-c),$$

while the equations for the Lorenz system are

43 $$\dot{x}	= \, \sigma (y-x),\\ \dot{y}	= \, x(\rho -z)-y,\\ \dot{z}	= \, xy-\beta z,$$

In both cases, the parameters are fixed so as the resulting dynamics is chaotic. Namely, for the Rössler oscillator we selected *a* = *b* = 0.2, *c* = 9, and for the Lorenz system *σ* = 10, *ρ* = 28, and *β* = 8/3.

Furthermore, as a real-world example, we have considered the HR model for the neuron, whose isolated dynamics is described by the set of equations

44 $$\dot{x}	= \, y+3{x}^{2}-{x}^{3}-z+I,\\ \dot{y}	= \, 1-5{x}^{2}-y,\\ \dot{z}	= \, -rz+rs(x+1.6),$$

Here we fixed *r* = 0.006, *s* = 4, *I* = 3.2, so that the resulting dynamics is chaotic^64^.

In all cases, the state of the system is monitored by the average synchronization error defined as

45 $$E={\left\langle {\left(\frac{1}{N(N-1)}\mathop{\sum }\limits_{i,j = 1}^{N}\parallel {{\bf{x}}}_{j}-{{\bf{x}}}_{i}{\parallel }^{2}\right)}^{\frac{1}{2}}\right\rangle }_{T},$$

where *T* is a sufficiently large window of time where the synchronization error is averaged, after discarding the transient.

Numerical integrations of the simplicial complexes of chaotic units are performed by means of an Euler algorithm, with integration step *δt* = 10^−4^, in a windows of time equal to 2*T* with *T* = 500.

For the calculation of the maximum Lyapunov exponent of the transverse modes in Eq. (3) we used the algorithm reported in ref. ^79^ (pp. 116–117) with the following parameters: integration step size *δt* = 10^−3^, number of iterations per cycle *I* = 10000, number of cycles *C* = 5.

For the calculation of the MSF (15) we made use of the algorithm for the computation of the entire spectrum of Lyapunov exponents in ref. ^80^ (with parameters: integration step size of the Euler algorithm *δt* = 10^−5^, length of the simulation *L* = 2500, windows of averaging *T* = 0.9*L*).

## Supplementary information

Peer Review File

## Data Availability

All data needed to evaluate the conclusions in the paper are present in the paper itself. Additional data related to this paper may be requested to the corresponding authors.

## Code availability

The code for the numerical simulations presented in this article is available from the corresponding authors upon reasonable request.
