# Supplementary material for: Stability of synchronization in simplicial complexes
Source: Nat Commun. 2021 Feb 23;12:1255. doi: 10.1038/s41467-021-21486-9 (PMC7902853; doi:10.1038/s41467-021-21486-9)
Supplement: Supplementary file 1 — Peer Review File [file 41467_2021_21486_MOESM1_ESM.pdf]

Reviewer #1 (Remarks to the Author):

In their manuscript “The Master Stability Function for Synchronization in Simplicial Complexes” the authors treat the stability of the synchronized state of an ensemble of identical network-coupled dynamical units where the network structure is assume to include both pairwise interactions (i.e., typical, 1-simplex connections) as well as higher-order interactions that are three-way, four-way, etc. (i.e., 2-, 3-, etc. simplex connections). (The main results, for purposes of simplicity, are focused on the case of networks with 1- and 2-simplex connections, but the authors discuss the inclusion of even higher-order connections.) The authors take an approach that is similar (in many ways is a generalization) to the original MSF framework where the stability of modes transverse to the the synchronization manifold are probed. Specifically, the largest Lyapunov exponent describing the growth/decay of perturbations along these transverse directions is calculated. The manuscript is well written and represents a strong step forward in terms of the theoretical treatment of network-coupled dynamical systems on simplicity complexes, particularly given the strong interest in simplicity complexes and higher-order interactions that currently resides in the network science community. It was a pleasure to read (thanks!) and I believe that it will be of significant interest to a wide audience of researchers working on networks and/or nonlinear dynamics. However, I have two large concerns of the current manuscript, detailed below. The first concern is somewhat technical and has to do with the nomenclature and description of the results as well as the actual scope of the results. The second concern has to do with the motivation or applicability of the work. I believe that to merit publication in Nature Communications these issues need to be addressed. Thus, in its current form I cannot recommend publication, but with appropriate revision this judgement may be reconsidered.

The first main issue I raise has to do with the description of the results themselves. In particular, as the authors point out, in the end, the case of simplicity complexes differs from the original MSF approach on a typical network in that the modes transverse to the synchronization manifold do not decouple. This causes several issues. First, there is a significant loss in the reduction of dimensionality - a system of N-1 equations need to be simulated as the transverse modes are intertwined, offering in some cases, essentially no reduction in dimensionality. (Although in other cases this not an insignificant reduction in dimensionality, but the system is still likely quite high dimensional.) Second, while it is the largest Lyapunov exponent of the resulting system that is the “MSF” in this case, it seems to me that calling this a MSF in the same sense as, for instance, the original work, is somewhat misleading. It seems to me that the authors have partially acknowledged this, as they refer to cases where this formalism is “identical to the classical MSF”, but nonetheless still advertise the result as the MSF for simplicity complexes, so the overall message is a bit confusing. In the authors’ view, what exactly in the current manuscript qualifies as a MSF? The full general case, or only the cases where the relevant modes decouple? It’s crucial to the overall message that the authors be unequivocal and precise about this.

The second issue I raise has to do with the motivation and applicability of the work. First, the motivation given for the presented results are somewhat lacking. The authors do a good job of motivated the need for a better understanding of (i) simplicial complexes from a network science point of view and (ii) synchronization phenomena in general, but little detail is given as to the combination of these general topics. In other words, what is the motivation for studying, specifically, identical dynamical units coupled via a simplicity complex? As the authors point out, strong motivation exists for phase (i.e., Kuramoto) oscillators. As far as I can see from the existing literature, this is for two reasons (although there may be more that I am missing at this point in time!): (i) the explicit derivation of higher-order coupling terms (from phase reductions of limit cycle oscillators) in phase oscillator systems and (ii) empirical evidence of simplicial complex structure in brain dynamics (where phase oscillators serve as simple models or various types of neurons and can be mapped to integrate-and-fire type oscillators). Can such strong motivations be pointed to for the current work? That is, (i) are there cases where higher-order interactions can be explicitly derived? And/or, (ii) are there classes of neuronal oscillators/dynamical systems where the current results can be applied to highlight novel collective phenomena? (Or are there any other strong motivations or applications of the current work

that may reside outside of these two possibilities?) In my opinion, while a very specific application of the current work is not necessarily required (especially given the new and quick-changing nature of simplicity complexes in the network science community), a broader range of potential areas of application, i.e., motivation, is needed for publication in Nature Communications and will strengthen the manuscript and its eventual impact on the network science and nonlinear dynamics communities significantly.

Reviewer #2 (Remarks to the Author):

This manuscript is an interesting contribution to the literature on generalization the classical master stability function to the simplicial complex which was a lack so far in the literature. The presentation of the method is very clear and one can follow it well.

And the generality of the title seems to indicate that the authors have solved all the problem of extending the master stability function to simplicial complexes . But in fact it is only valid on two very particular situations where the topology of the connectivity structure is an all-to-all coupling , or the natural coupling with the condition (10). These are very strong limitations, thus the method can only apply to very limited scenarios.

Moreover, in the paper, the authors do not make any specific assumption except for the fact that the system dynamics have to be identical. But in the ordinary case, through the proposed method, we may need to calculate the maximum Lyapunov exponent for a system of N-1 coupled linear equations, where N is the number of the node in simplicial complex. Hence, there is the high computational complexity of the method proposed in this paper, especially if the size of the simplicial complexes becomes large.

Although the model (1) is typical, I would like to see an instructive real world example included in detail. At the same time, taking 2-dimensional simplicial complexe as an example, I miss whenever the triangle can be considered as a 2-simplex or only as three pairwise interactions in a real-word example.

In Fig.1, from the sketches of the simplicial complexes, one can not clearly observe that whether a triangle is effectively taken into consideration as a 2-simplex.

Reviewer #3 (Remarks to the Author):

The reviewer found this paper a solid contribution to the literature focused on the study of synchronization via the Master Stability Function. I believe that the technical results are correct. My main comments are therefore related to the scope and impact of the presented results. In particular:

1. the authors motivate in different parts of the manuscript their work with statements such as "Various systems in physics, biology, social sciences and engineering found a proper representation as networks of coupled dynamical systems, where the graph links describe pairwise interactions. This is, however, too strong a limitation, as recent studies have revealed that higher-order many-body interactions are present in social groups, ecosystems and in the human brain, and they actually affect the emergent dynamics of all these systems". Following this motivation, the reviewer would have expected a more concrete application of the result, for example coming from neuronal networks, epidemics dynamics, ... which are so important nowadays;

2. overall, I found the results abstract and I believe that simple simulations that make use of chaotic

oscillators do not help in understanding the impact of the results. I believe the authors should use their technique to discuss a real-world application. I would expect a NatComms paper to have, besides a solid mathematical machinery, a real-world impact. For examples, as a suggestion, the authors could apply their techniques to study neuronal dynamics or epidemic dynamics, for which we have today several data. Using the results, can the authors obtain insides on these (or other, chosen by the authors) applications?

3. the structure of the paper would also need to be improved. I recommend the authors to split the paper in a main body (that goes straight to the point to the implications and impact of the results) and supplementary information (that gives the mathematical details and proofs).

4. I understand, as stated also by the authors that only paradigmatic examples are considered in the manuscript. What is not clear to me is the limitations of the approach. For example, what would take to embed in this framework also cluster-synchronization phenomena?

Resubmission of Manuscript NCOMMS-20-34385
Stability of Synchronization in Simplicial Complexes
(previously submitted as "The Master Stability Function for Synchronization in Simplicial Complexes")

by L.V. Gambuzza, F. Di Patti, L. Gallo, S. Lepri, M. Romance, R. Criado, M. Frasca, V. Latora and S. Boccaletti

Dear Referees,

Thank you very much for your comments on our manuscript: "The Master Stability Function for Synchronization in Simplicial Complexes". Please, find enclosed a point-to-point reply to your indications, in which we refer to the comments in bold faces, while our responses are provided in regular text. The corresponding changes made to the manuscript are shown in red.

We thank you in advance for the attention that you will dedicate to our resubmission, and we take the occasion for sending you our best regards

Lucia Valentina Gambuzza
Francesca Di Patti
Luca Gallo
Stefano Lepri
Miguel Romance
Regino Criado
Mattia Frasca
Vito Latora
Stefano Boccaletti

RESPONSES TO REFEREE 1

In their manuscript "The Master Stability Function for Synchronization in Simplicial Complexes" the authors treat the stability of the synchronized state of an ensemble of identical network-coupled dynamical units where the network structure is assume to include both pairwise interactions (i.e., typical, 1-simplex connections) as well as higher-order interactions that are three-way, four-way, etc. (i.e., 2-, 3-, etc. simplex connections). (The main results, for purposes of simplicity, are focused on the case of networks with 1- and 2-simplex connections, but the authors discuss the inclusion of even higher-order connections.) The authors take an approach that is similar (in many ways is a generalization) to the original MSF framework where the stability of modes transverse to the the synchronization manifold are probed. Specifically, the largest Lyapunov exponent describing the growth/decay of perturbations along these transverse directions is calculated. The manuscript is well written and represents a strong step forward in terms of the theoretical treatment of network-coupled dynamical systems on simplicity complexes, particularly given the strong interest in simplicity complexes and higher-order interactions that currently resides in the network science community. It was a pleasure to read (thanks!) and I believe that it will be of significant interest to a wide audience of researchers working on networks and/or nonlinear dynamics. However, I have two large concerns of the current manuscript, detailed below. The first concern is somewhat technical and has to do with the nomenclature and description of the results as well as the actual scope of the results. The second concern has to do with the motivation or applicability of the work. I believe that to merit publication in Nature Communications these issues need to be addressed. Thus, in its current form I cannot recommend publication, but with appropriate revision this judgement may be reconsidered.

We thank Referee 1 for the efforts he/she dedicated to review our manuscript, and the positive evaluation of our paper. In the following we provide a point-to-point answer to the raised concerns.

FIRST ISSUE. The first main issue I raise has to do with the description of the results themselves. In particular, as the authors point out, in the end, the case of simplicity complexes differs from the original MSF approach on a typical network in that the modes transverse to the synchronization manifold do not decouple. This causes several issues. First, there is a significant loss in the reduction of dimensionality - a system of N-1 equations need to be simulated as the transverse modes are intertwined, offering in some cases, essentially no reduction in dimensionality. (Although in other cases this not an insignificant reduction in dimensionality, but the system is still likely quite high dimensional.) Second, while it is the largest Lyapunov exponent of the resulting system that is the "MSF" in this case, it seems to me that calling this a MSF in the same sense as, for instance, the original work, is somewhat misleading. It seems to me that the authors have partially acknowledged this, as they refer to cases where this formalism is "identical to the classical MSF", but nonetheless still advertise the result as the MSF for simplicity complexes, so the overall message is a bit confusing. In the authors' view, what exactly in the current manuscript qualifies as a MSF? The full general case, or only the cases where the relevant modes decouple? It's crucial to the overall message that the authors be unequivocal and precise about this.

The Referee's comment is particularly important as in our paper we have shown that simplifying the stability problem into a single parametric variational equation is enabled only by special cases, whereas the simplicial complex framework does not allow in general such simplification. This fact points out that in the more general case the simplicial complex cannot be reduced in any way to a connectivity pattern assimilable to a complex network, but, on the contrary, it is a different (and more intricate) structure requiring a tailored method for the stability analysis of synchronization. At the same time, it is important to remark that the result achieved ultimately relies on the computation of a single quantity, representing the maximum Lyapunov exponent of the transverse modes, calculated on a system of linear equations, and as such is effective also from the computational point of view.

To make more clear our message, we have now emended the text in several points and, accordingly, also changed the title of the paper in "Stability of Synchronization in Simplicial Complexes". The different scenarios have been also dealt with in different sections of the manuscript.

SECOND ISSUE: The second issue I raise has to do with the motivation and applicability of the work. First, the motivation given for the presented results are somewhat lacking. The authors do a good job of motivated the need for a better understanding of (i) simplicial complexes from a network

science point of view and (ii) synchronization phenomena in general, but little detail is given as to the combination of these general topics. In other words, what is the motivation for studying, specifically, identical dynamical units coupled via a simplicity complex? As the authors point out, strong motivation exists for phase (i.e., Kuramoto) oscillators. As far as I can see from the existing literature, this is for two reasons (although there may be more that I am missing at this point in time!): (i) the explicit derivation of higher-order coupling terms (from phase reductions of limit cycle oscillators) in phase oscillator systems and (ii) empirical evidence of simplicial complex structure in brain dynamics (where phase oscillators serve as simple models or various types of neurons and can be mapped to integrate-and-fire type oscillators). Can such strong motivations be pointed to for the current work? That is, (i) are there cases where higher-order interactions can be explicitly derived? And/or, (ii) are there classes of neuronal oscillators/dynamical systems where the current results can be applied to highlight novel collective phenomena? (Or are there any other strong motivations or applications of the current work that may reside outside of these two possibilities?) In my opinion, while a very specific application of the current work is not necessarily required (especially given the new and quick-changing nature of simplicity complexes in the network science community), a broader range of potential areas of application, i.e., motivation, is needed for publication in Nature Communications and will strengthen the manuscript and its eventual impact on the network science and nonlinear dynamics communities significantly.

We fully agree with the Referee's considerations, and we have now explicitly mentioned in the introduction the application of our general framework to neuron dynamics. In this context, several works have pointed out an important role of higher-order interactions in synchronous neuronal activity, but the problem of how to model them in a nonlinear dynamics framework is still open. In the new version of the text we have also introduced a case study showing the suitability of our approach that we expect to be of practical use once the question of the model of the higher-order interactions will be fully addressed by neuroscientists.

Other fields of study where our approach can be applied are ecological systems and nonlinear consensus, that have been now cited in our Manuscript. In all these cases, often the oscillators are considered to be identical, at least at first approximation. This hypothesis is quite important to develop the calculations for the stability analysis, although several works have demonstrated that the results often extend in case of parameter mismatches.

In summary, we would like to express our deepest gratitude to this Referee, as we are really convinced that his/her suggestions and remarks have greatly contributed to improve the quality and clarity of our presentation.

We feel confident to have fulfilled all the Referee's concerns, and we hope that Referee 1 will now find our revised version suitable for publication.

RESPONSES TO REFEREE 2

This manuscript is an interesting contribution to the literature on generalization the classical master stability function to the simplicial complex which was a lack so far in the literature. The presentation of the method is very clear and one can follow it well.

First of all, we would like to thank Referee 2 for the time and efforts devoted in his/her careful revision of our Manuscript, and for the positive evaluation of the presentation of our results.

And the generality of the title seems to indicate that the authors have solved all the problem of extending the master stability function to simplicial complexes. But in fact it is only valid on two very particular situations where the topology of the connectivity structure is an all-to-all coupling , or the natural coupling with the condition (10). These are very strong limitations, thus the method can only apply to very limited scenarios. Moreover, in the paper, the authors do not make any specific assumption except for the fact that the system dynamics have to be identical. But in the ordinary case, through the proposed method, we may need to calculate the maximum Lyapunov exponent for a system of N-1 coupled linear equations, where N is the number of the node in simplicial complex. Hence, there is the high computational complexity of the method proposed in this paper, especially if the size of the simplicial complexes becomes large.

The Referee has correctly pointed out an ambiguity deriving from the title of our Manuscript that has now been corrected in "Stability of Synchronization in Simplicial Complexes" to make clear the contribution of our work. Indeed, our results solve the problem of stability for synchronization in the general case, but, as correctly noticed by Referee 2, this problem can be formulated in terms of a Master Stability Function only in the all-to-all and natural coupling cases. This observation crucially remarks that in the more general case a simplicial complex cannot be reduced in any way to a connectivity pattern assimilable to a complex network, but, on the contrary, it is a different (and more intricate) structure requiring a tailored method for the stability analysis of synchronization. At the same time, it is important to remark that the result achieved ultimately relies on the computation of a single quantity, representing the maximum Lyapunov exponent of the transverse modes, calculated on a system of linear equations, and as such is effective also from the computational point of view.

In conclusion, the point is not that the problem of the stability of synchronization has not been solved in terms of a Master Stability Function, but that it cannot be formulated as such. We have, on the contrary, highlighted which conditions enable this formalism and provided a solution to the problem of stability of synchronization in the more general case.

To make more clear the limitations of our manuscript (and the potentialities), we have now emended the text in several points, highlighted in red in the new version.

Although the model (1) is typical, I would like to see an instructive real world example included in detail. At the same time, taking 2-dimensional simplicial complexe as an example, I miss whenever the triangle can be considered as a 2-simplex or only as three pairwise interactions in a real-word example.

The comment from Referee 2 on the need for a real-world example is of great relevance. Following Referee 2's suggestion, in the new version of the manuscript we have presented and discussed an example of neuronal dynamics. In particular, we have analyzed a network of Hindmarsh-Rose neurons, subject not only to pairwise coupling but also to three-bodies interactions, finding good agreement between our theoretical predictions and numerical simulations. To the best of our knowledge, the inclusion of higher-order interactions in these models is biologically very relevant, as recent evidences in neuroscience have pointed out their existence in neuron networks. In fact, incorporating higher-order coupling permits to model the effect of astrocytes and other glial cells, that are considered a plausible source of high-order interactions as they play a fundamental role in modulating the synaptic function. In particular, individual astrocytes can make contact with thousands of synapses, meaning that "synapses don't consist of just a pre- and postsynaptic neuronal element, but that many also have an astrocytic projection that envelops the synapse" (see Ref. 65 of the main text). Given the importance of synchronization in neural networks, as it is associated to epileptic phenomena, we believe that our work can pave the way to further studies on seizures.

In Fig.1, from the sketches of the simplicial complexes, one can not clearly observe that whether a triangle is effectively taken into consideration as a 2-simplex.

Referee 2 is correct. In our first version of the Manuscript we have not properly indicated the presence of 2-simplices in the structure. We have now used a clearer representation of these structures, adopted in several other papers, where 2-simplices are highlighted with a shadowed area.

In summary, we are grateful to this Referee, because his/her suggestions and remarks have contributed to improve both quality and clarity of our presentation. We feel confident to have fulfilled all the Referee's concerns, and we hope that Referee 2 will now find our revised version suitable for publication.

RESPONSES TO REFEREE 3

The reviewer found this paper a solid contribution to the literature focused on the study of synchronization via the Master Stability Function. I believe that the technical results are correct. My main comments are therefore related to the scope and impact of the presented results. In particular:

1. the authors motivate in different parts of the manuscript their work with statements such as "Various systems in physics, biology, social sciences and engineering found a proper representation as networks of coupled dynamical systems, where the graph links describe pairwise interactions. This is, however, too strong a limitation, as recent studies have revealed that higher-order many-body interactions are present in social groups, ecosystems and in the human brain, and they actually affect the emergent dynamics of all these systems". Following this motivation, the reviewer would have expected a more concrete application of the result, for example coming from neuronal networks, epidemics dynamics, ... which are so important nowadays;

We thank Referee 3 for the comment. Following Referee 3's suggestion, in the current version of the manuscript we have presented and discussed the important example of neuronal dynamics (please, also see our reply to the next point for a further discussion on this issue).

2. overall, I found the results abstract and I believe that simple simulations that make use of chaotic oscillators do not help in understanding the impact of the results. I believe the authors should use their technique to discuss a real-world application. I would expect a NatComms paper to have, besides a solid mathematical machinery, a real-world impact. For examples, as a suggestion, the authors could apply their techniques to study neuronal dynamics or epidemic dynamics, for which we have today several data. Using the results, can the authors obtain insides on these (or other, chosen by the authors) applications?

The comment from Referee 3 on the need for a real-world example is of great relevance. As mentioned in the previous point, in the new version of the Manuscript we have now considered an example of neuronal dynamics. In particular, we have analyzed a network of Hindmarsh-Rose neurons, subject not only to pairwise coupling but also to three-bodies interactions, finding good agreement between theoretical predictions and numerical simulations. To the best of our knowledge, the inclusion of higher-order interactions in these models is biologically very relevant, as recent evidences in neuroscience have pointed out their existence in neuron networks. In fact, incorporating higher-order coupling permits to model the effect of astrocytes and other glial cells, that are considered a plausible source of high-order interactions as they play a fundamental role in modulating the synaptic function. In particular, individual astrocytes can make contact with thousands of synapses, meaning that "synapses don't consist of just a pre- and postsynaptic neuronal element, but that many also have an astrocytic projection that envelops the synapse" (see Ref. 65 of the main text). Given the importance of synchronization in neural networks, as it is associated to epileptic phenomena, we believe that our work can pave the way to further studies on seizures.

3. the structure of the paper would also need to be improved. I recommend the authors to split the paper in a main body (that goes straight to the point to the implications and impact of the results) and supplementary information (that gives the mathematical details and proofs).

Following the Referee's recommendation, we have now improved the structure of the paper by moving most of the mathematical derivations to the methodological section, thus emphasising the results obtained and stating them in a more concise way.

4. I understand, as stated also by the authors that only paradigmatic examples are considered in the manuscript. What is not clear to me is the limitations of the approach. For example, what would take to embed in this framework also cluster-synchronization phenomena?

Once again, we thank Referee 3 for this comment. The framework we consider is general and, although we have focused on complete synchronization, the study of other patterns of synchronization that may arise in such structure is really intriguing. This is particularly true for the example mentioned by Referee 3. Although extending the notion of cluster synchronization to simplicial complexes requires a formal definition of symmetries in the general framework of multi-body interactions, that is beyond the purpose of our manuscript, in the new version of the Manuscript we have incorporated the suggestion of the Referee by illustrating an example of a simplicial complex displaying cluster

synchronization. In particular, we have highlighted how two structures having the same set of 1-simplices, but different 2-simplices, may display a totally different behavior in terms of cluster synchronization.

Finally, we would like to express our deep gratitude to Referee 3, for her/his suggestions and remarks that have greatly contributed to improve the quality and clarity of our presentation. We feel confident to have fulfilled all the Referee's concerns, and we hope that Referee 3 will now find our revised version suitable for publication.

Reviewer #1 (Remarks to the Author):

In their revised manuscript the authors appear to made some improvements to their initial submission. Regarding the first of my two main points from my initial report, the manuscript seems to have been adequately addressed. In particular, the changes make the message much more accurate, in that a true MSF framework is not completely described in general. For the second point, the authors have included some results using a neuronal model, which makes sense given the likely prevalence of higher-order interactions in brain dynamics. I also appreciate the inclusion of tanh(…) coupling - I think that this actually adds to the theory as an example of handling a different kind of nonlinear coupling.

In light of these modifications, I think the following points need to be kept in mind. First, as the authors have now better described from the title and onwards, the theory presented here applies to a non-complete picture of synchronization phenomena in simplicial complexes, given the limitations addressed in the paper. Second, while the model chosen to highlight the application of the theory makes sense in that it is a neuronal model (i.e., there is some connection to brain dynamics), it is still just a model and the higher-order interactions between different elements have just been chosen to make sense mathematically. In other words, it's not clear that actual neurons or other elements related to brain dynamics experience coupling in this or other similar ways. (I want to again contrast this with the case of phase dynamics where rigorous methods have been applied to extract what the precise nature of coupling is at different orders.)

Let me still emphasize that I believe the work is very nice and will be very useful, but given the limitations above, in addition to the fact that truly new physics or dynamical phenomena are not discovered here, the usefulness and impact of this work will likely be restricted primarily to the physics community. For this reason, I believe that while the paper should not be published in Nature Communications, it should be in a more physics-oriented journal, whether it be transferred internally to Communications Physics or externally to, say PRR.

Reviewer #2 (Remarks to the Author):

The manuscript "Stability of Synchronization in Simplicial Complexes" is an interesting contribution to the literature on synchronization in Simplicial Complexes. The authors have addressed my previous concerns. However, I still have the following suggestions/concerns.

1.The presentation of this subtle method is very clear and one can follow it well. However, the structure of the paper may need to be improved to make it easier to understand.

2.As for the computational complexity of the method proposed in this paper, one only need to calculate the Lyapunov exponents of the N-1 coupled linear equations. However at the same time, only the linear stability for synchronization can be studied. Then whether the global stability for synchronization in Simplicial Complexes can also be obtained by other methods, such as the basin stability (BS) measurement.

3.For the numerical simulations, the authors take the high-order Hindmarsh-Rose neurons and the Zachary karate club as very paradigmatic examples. For Zachary karate club, I wonder what the three-body interactions and the variation of the percentage of the three-body interactions indeed indicate in the reality. And I recommend that the authors should make clear that they are using examples that could not be addressed before they came up with their proposal.

Reviewer #3 (Remarks to the Author):

All my previous comments have been addressed but my main criticism in the rather-abstract setting of the results is still confirmed.
